# Exploration of potential biomarkers and immune cell infiltration characteristics for peripheral atherosclerosis in sjögren’s syndrome based on comprehensive bioinformatics analysis and machine learning

**DOI:** 10.3389/fgene.2025.1546315

**Published:** 2025-07-30

**Authors:** Chunjiang Liu, Yuan Wang, Lina Zhou, Feifei Cai, Xiaoqi Tang, Liying Wang, Xiang Zhang

**Affiliations:** ^1^ Department of General Surgery, Division of Vascular Surgery, Shaoxing People’s Hospital (The First Affiliated Hospital, Shaoxing University), Shaoxing, China; ^2^ Department of Intervention Vascular, Hefei Hospital Affiliated to Anhui Medical University, Hefei, China; ^3^ Department of Anesthesiology, Shaoxing People’s Hospital (The First Affiliated Hospital, Shaoxing University), Shaoxing, China; ^4^ Department of Radiology, Shaoxing People’s Hospital (The First Affiliated Hospital, Shaoxing University), Shaoxing, China; ^5^ Department of General Surgery, Wuxi No.2 People’s Hospital (Jiangnan University Medical Center), Wuxi, China

**Keywords:** peripheral atherosclerosis, Sjögren’s syndrome, biomarkers, immune infiltration, bioinformatics analysis, machine learning

## Abstract

**Background:**

Sjögren’s syndrome (SS) is an autoimmune disorder impacting exocrine glands, while peripheral atherosclerosis (PA) demonstrates a close link to inflammation. Despite a notable rise in atherosclerosis risk among SS patients in prior investigations, the precise mechanisms remain elusive.

**Methods:**

A comprehensive analysis was conducted on seven microarray datasets (GSE7451, GSE23117, GSE143153, GSE28829, GSE100927, GSE159677, and GSE40611). The LIMMA package, in conjunction with weighted gene co-expression network analysis (WGCNA), provides a robust method for identifying differentially expressed genes (DEGs) associated with peripheral atherosclerosis (PA) in Sjögren’s syndrome (SS). Subsequently, machine learning algorithms and protein-protein interaction (PPI) network analysis were employed to further investigate potential predictive genes. These findings were utilized to construct a nomogram and a receiver operating characteristic (ROC) curve, which assessed the predictive accuracy of these genes in PA patients with SS. Additionally, extensive analyses of immune cell infiltration and single-sample gene set enrichment analysis (ssGSEA) were conducted to elucidate the underlying biological mechanisms.

**Results:**

Using the LIMMA package and WGCNA, 135 DEGs associated with PA in SS were identified. PPI network analysis revealed 17 candidate hub genes. The intersection of gene sets identified by three distinct machine learning algorithms highlighted CCL4, CSF1R, and MX1 as key DEGs. ROC analysis and nomogram construction demonstrated their high predictive accuracy (AUC: 0.971, 95% CI: 0.941–1.000). Analysis of immune cell infiltration showed a significant positive correlation between these hub genes and dysregulated immune cells. Additionally, ssGSEA provided critical biological insights into the progression of PA in SS.

**Conclusion:**

This study systematically identified three promising hub genes (CCL4, CSF1R, and MX1) and developed a nomogram for predicting PA in SS. Analysis of immune cell infiltration demonstrated that dysregulated immune cells significantly contribute to the progression of PA. Additionally, ssGSEA analysis offered important insights into the mechanisms by which SS leads to PA.

## 1 Introduction

Sjögren’s syndrome (SS) is characterized by the immune system’s response to self-tissue antigens, resulting in histopathological damage. This process triggers a chronic inflammatory condition that affects various organs and tissues. Peripheral atherosclerosis (PA) primarily impacts the lower extremities and carotid arteries, potentially leading to lower limb ischemia, necrosis, and cerebrovascular events. It is well known that the occurrence of atherosclerosis is based on the interaction of traditional cardiovascular risk factors (CRF), inflammatory events and immune mechanisms (Safar, 2018). Recent studies have underscored the significant association between SS and atherosclerosis. The potential mechanisms linking these conditions likely involve systemic inflammation and endothelial dysfunction, common features of autoimmune disorders like SS (Gravani et al., 2015). Research indicates that patients with SS have impaired endothelial function, which may be the initiating step in the formation of atherosclerosis (Kiripolsky and Kramer, 2018). Endothelial cells are pivotal in atherosclerosis pathogenesis, leading to the increased expression of adhesion molecules such as intercellular adhesion molecule 1 (ICAM-1) and vascular cell adhesion molecule 1 (VCAM-1). These molecules, serving as biomarkers for endothelial injury, exhibit significant elevation in individuals with SS, suggesting a plausible association between SS and PA (Liu et al., 2023). In SS patients, there is a positive correlation between the intima-media thickness of the carotid and femoral arteries and the level of anti-Sjögren’s syndrome antibody A (SSA) in the bloodstream (O et al., 2019; Turesson et al., 2004). This correlation may stem from the autoimmune response triggering vascular wall inflammation and hastening atherosclerosis progression through interactions among various inflammatory cells and cytokines. Immune cells are recruited and migrate to the vascular wall through adhesion molecules (Zheng et al., 2009), and the pro-inflammatory microenvironment of SS further promotes this pathological process (Nair et al., 2012). Atherosclerosis shares similar inflammatory mediators and immune mechanisms with autoimmune rheumatic diseases. Due to the over-activation of the immune system and chronic inflammatory state, patients with SS have a potential pathological process that can accelerate the progression of atherosclerosis.

Bartoloni et al. conducted a retrospective analysis on 1343 SS patients, unveiling a significantly increased risk of cardiovascular and cerebrovascular diseases (Bartoloni et al., 2015). Another prospective cohort study indicated that the carotid intima-media thickness thickening in SS patients occurred earlier and faster than in the control group, especially after the age of 50, and the likelihood of plaque formation was also notably higher in this patient population (Zehrfeld et al., 2024). A study involving 155 SS patients showed that the prevalence of atherosclerosis (AS) was 41.3%, with major cardiovascular and cerebrovascular events occurring at a rate of 5.2% (Liu et al., 2023). The diagnosis of SS complicated by PA is challenging because of the absence of typical clinical symptoms in the early stage and the risk of missed diagnosis due to the limitations of imaging examinations such as ultrasonography, which has insufficient sensitivity to early lesions. Given the rising interest in the connection between SS and atherosclerosis as prevalent autoimmune disorders, identifying common biomarkers for SS and PA and investigating their shared pathogenesis is crucial. These research findings hold promise for early detection and intervention in SS and PA cases. Timely diagnosis and management of PA in SS individuals are imperative for minimizing adverse outcomes and improving patient prognosis.

In recent years, the application of microarray gene expression profiling has emerged as a powerful technique for identifying biomarkers, showing promise in the realm of SS and arteriosclerosis. Numerous studies have shown that there are multiple relationships between the onset of SS and various genetic and environmental factors (Miceli-Richard and Criswell, 2014). The saliva of Sjögren’s syndrome patients may contain multiple biomarkers, including soluble siglec-5, which could potentially correlate with disease severity (Lee et al., 2019). As regard to arteriosclerosis, research has pinpointed protein tyrosine phosphatase receptor type J (PTPRJ) and dehydrogenase/reductase 9 (DHRS9) as pivotal biomarkers with high diagnostic accuracy for the pathological process of atherosclerosis (Xu et al., 2022). Autoimmune responses in Sjögren’s syndrome instigate inflammation within the vascular wall, intensifying atherosclerosis progression. Consequently, biomarkers linked to immune functionality might emerge as crucial predictive indicators for the predisposition of Sjögren’s syndrome patients to atherosclerosis, potentially informing treatment decisions. The scarcity of literature addressing the specific genetic mechanisms underlying Sjögren’s syndrome-induced atherosclerosis underscores the imperative for further investigation. While the broad utility of microarray technology in these disease realms is evident, the precise pathogenesis of these conditions remains enigmatic, necessitating additional research to unravel their specific pathological mechanisms.

Bioinformatics technologies hold significant potential for elucidating disease mechanisms and identifying biological markers (Zhou et al., 2022; Zhu et al., 2023; Wang et al., 2022). Limma analysis proficiently manages gene chip data, discerns differentially expressed genes, and plays a pivotal role in investigating disease mechanisms and identifying biological markers. WGCNA correlates gene modules with phenotypes through the construction of gene co-expression networks, with its central genes serving as vital biological indicators. PPI analysis scrutinizes protein interactions using databases and experimental data, facilitating the comprehension of disease mechanisms and identification of crucial protein markers. Machine learning delves into disease mechanisms, selecting genes with high predictive value as markers by analyzing data patterns with algorithms. The construction and analysis of a nomogram merge diverse predictive variables to yield a comprehensive risk score, aiding in the identification of potential predictive and diagnostic markers. Immune cell infiltration analysis examines the immune cell status in affected tissues, a crucial aspect in unveiling disease immune mechanisms and identifying pertinent markers. These technologies offer practical utility in exploring the specific genetic mechanisms triggered by SS for PA. Integrating bioinformatics and machine learning methods enables the discovery of potential disease mechanisms and identification of biomarkers (Vaziri-Moghadam and Foroughmand-Araabi, 2024).

## 2 Methods

### 2.1 Microarray acquisition and data processing

Using the keywords “Sjögren’s syndrome” or “peripheral atherosclerosis,” seven microarray datasets (GSE7451, GSE23117, GSE143153, GSE28829, GSE100927, GSE159677, GSE40611) were screened from the NCBI Gene Expression Omnibus (GEO) (Clough and Barrett, 2016). Detailed information regarding these datasets is presented in Table 1. The GSE7451 (Hu et al., 2007), GSE23117 (Greenwell-Wild et al., 2011), GSE143153 (Joachims et al., 2020), and GSE40611 (Horvath et al., 2012) datasets contain gene expression data from individuals with Sjögren’s syndrome and normal controls. The GSE100927 (Steenman et al., 2018) dataset includes gene expression profiles from 69 human peripheral arteries affected by atherosclerosis (including carotid arteries, femoral arteries, and popliteal arteries) and 35 control arteries without atherosclerotic lesions. The GSE159677 (Alsaigh et al., 2022) dataset contains single-cell transcriptome profiles from three calcified atherosclerotic core (AC) plaques and three controls (patient-matched proximal adjacent regions of the carotid artery). The GSE28829 dataset was used for external validation.

**TABLE 1:** Basic information of GEO datasets used in the study.

ID	GSE series	Disease	Samples	Source Types	Platform	Group
1	GSE7451	SS	10 SS patients and 10 normal controls	salivary gland	GPL570	Discovery cohort
2	GSE23117	SS	11 SS patients and 4 normal controls	salivary gland	GPL570	Discovery cohort
3	GSE143153	SS	17 SS patients and 15 normal controls	salivary gland	GPL13607	Discovery cohort
4	GSE100927	PA	69 peripheral atherosclerotic patients and 35 controls	Arterie	GPL17077	Discovery cohort
5	GSE159677	PA	3 carotid plaque patients and 3 matched proximal adjacent portion patients	Arterie	GPL18573	Discovery cohort
6	GSE28829	PA	16 advanced carotid plaque patients and 13 early carotid plaque patients	Arterie	GPL570	Validation cohort
7	GSE40611	SS	18 SS patients and 17 normal controls	Parotid gland	GPL570	Validation cohort

We preprocessed the downloaded raw datasets using the “affy” R package from the Bioconductor project, including background adjustment, log2 transformation (for GSE7451 and GSE23117), and quantile normalization. When multiple probes matched the same gene, we used the median as the final expression measurement for that gene. After converting probes to gene symbols, we prepared matrix files. Subsequently, we merged the GSE7451, GSE23117, and GSE143153 datasets and used the R package Surrogate Variable Analysis (SVA) (Leek et al., 2012) to remove unwanted variations and batch effects. Batch effect correction was carried out using the ComBat function within the “sva” R package. In this process, the mod = model.matrix (∼group) parameter was utilized to designate the biological grouping as covariates, preserving biological variations associated with the grouping while eliminating batch effects. Following this, PCA visualization was employed to assess the efficacy of batch effect removal. The prcomp function in the stats package was used to perform PCA on the data before and after batch effect removal. The first principal component (PC1) and the second principal component (PC2) were visualized with the ggplot2 R package. In the principal component analysis, the Cumulative Variance Explained method was adopted as the selection criterion. That is, several top principal components were chosen such that their cumulative explained variance reached a certain threshold (typically 70%–90%), thereby achieving dimensionality reduction of high-dimensional data.

### 2.2 DEGs identification in SS and PA

Based on established criteria (P < 0.05 and fold change (FC) > 1.5), we utilized the “Limma” package to identify DEGs between SS and the control group in the merged dataset (GSE7451, GSE23117, and GSE143153), as well as DEGs between PA and the control group in the GSE100927 dataset. Utilizing heatmaps to illustrate the expression patterns of differentially expressed genes across various samples, and generating volcano plots to visually represent the distribution of these genes.

### 2.3 Significant module identification via WGCNA in SS and PA

WGCNA, a powerful strategy for constructing co-expression networks, has been widely utilized in large dataset analyses (Yang et al., 2020). We utilized WGCNA to identify gene modules significantly linked to SS and PA. In this study, the analysis process was as follows: Initially, we computed the absolute median deviation (MAD) for each gene expression value and removed the bottom 50% of genes with the lowest MAD values. Next, the goodSamplesGenes function was applied to filter the DEGs expression matrix and construct a scale-free co-expression network. Using co-expression similarity, the soft-thresholding parameter β was determined via the pickSoftThreshold function, which was used to convert gene correlation coefficients into a weighted adjacency matrix. Thereafter, the Topological Overlap Matrix (TOM), which offers an improved representation of gene co-expression relationships, was computed. Hierarchical clustering was then performed on the TOM to group genes with similar expression patterns into modules using dynamic tree cutting. To prevent over-segmentation, small modules with high similarity were merged based on a minimum module size threshold of 50 in the gene dendrogram. Finally, distinct modules were further analyzed through changes in estimated module eigengenes to identify those significantly correlated with the studied phenotypes, and a visualization of the eigengene network was generated.

### 2.4 Functional enrichment analysis

Kyoto Encyclopedia of Genes and Genomes (KEGG) (Ogata et al., 1999) serves as a pivotal knowledge repository for systematic gene function analysis. Within Gene Ontology (GO) analysis (Kuleshov et al., 2019), categories such as biological process (BP), cellular component (CC), and molecular function (MF) are delineated. In order to further uncover the physiopathological mechanisms of PA in SS patients. KEGG and GO functional enrichment analysis were conducted using the “ClusterProfiler” R package, with the corresponding top 10 GO terms in each category were visualized using the “ggplot2” R package (Yu et al., 2012). Screening criteria included a false discovery rate below 0.05, and adjusted P value lower than 0.05 is described as statistically significant.

### 2.5 PPI network construction and candidate hub genes selection

With a set minimum interaction score of 0.400, PPI network was constructed in STRING database (https://cn.string-db.org; version 12.0) (S et al., 2021). In practice, the genes that did not interact with each other were concealed. Following the download of the interaction data file, visual representation was accomplished using the Cytoscape software (Otasek et al., 2019). To analyze topology, the Cytoscape plug-in CytoHubba (Su et al., 2021) was utilized, employing a trio of distinct algorithms (degree, betweenness, closeness centrality). Ultimately, the intersection of these algorithms facilitated the visualization of the top 30 DEGs.

### 2.6 Machine-learning

In machine learning, the Support Vector Machine-Recursive Feature Elimination (SVM-RFE), Logistic Regression with Least Absolute Shrinkage and Selection Operator (LASSO), and Random Forest algorithms collectively facilitate the identification of potential gene biomarkers. The SVM-RFE (Huang et al., 2014) technique, within the Support Vector Machine framework, aimed to identify optimal variables by eliminating feature vectors. We employed 10-fold cross-validation in the SVM-RFE algorithm to reduce bias and ensure robust feature ranking. The FeatSweep.wrap function was utilized to train SVM models for each feature subset and evaluate their performance. Through the PlotErrors and Plotaccuracy functions, we visualized and identified the highest precision and lowest error rates. LASSO (Tibshirani, 1996) regression is commonly employed to mitigate overfitting in variable selection. It aims to attain the variables' outcomes with minimal prediction error and their corresponding regression coefficients, leading to optimal results. 10-fold cross-validation with the lowest standard was used to select the optimal parameter (lambda) in the LASSO model. We solved the coefficient of the gene and excluded the coefficient that is zero. Random Forest (Blanchet et al., 2020) was recognized for its ability to handle high-dimensional data, create predictive models, and evaluate variable significance. Finally, we performed an intersection analysis on the genes obtained from the three algorithms for further research.

### 2.7 ROC evaluation and nomogram construction

We examined the expression of each candidate gene in individuals with PA compared to the control group through the student's t-test. To assess the predictive and diagnostic potential of these genes, we plotted ROC curves and calculated their area under the curve (AUC) with a 95% confidence interval (CI). An AUC value surpassing 0.7 is deemed a suitable benchmark for predictive SS with PA. Additionally, we employed the “rms” package in R to generate a nomogram, assigning scores to genes based on their relative expression levels. By aggregating the scores per gene, we derived a cumulative score for predicting the risk of SS leading to PA. Lastly, an ROC curve was generated for the nomogram to showcase its predictive performance.

### 2.8 qRT-PCR of the hub genes and evaluation of the predictive model

To clinically validate the screened hub genes, a retrospective cohort analysis was conducted at Shaoxing People’s Hospital. The study enrolled patients admitted between 1 June 2024, and 10 June 2025, including 7 patients with SS and 7 patients with SS and PA. SS diagnosis followed the international criteria (Shiboski et al., 2017), whereas PA was confirmed by imaging evidence from color doppler ultrasound sonography or computed tomography angiography (CTA). Baseline clinical characteristics of the enrolled subjects are provided in Supplementary Table S1. This study was approved by the hospital’s Ethics Committee (Approval No.: 2025-Scientific Research Project 103-01). Following peripheral blood collection, total RNA was extracted using the MolPure^®^ Blood RNA Extraction Kit according to the manufacturer’s protocol. Subsequently, cDNA synthesis was performed using the Hifair^®^Ⅱ First-Strand cDNA Synthesis Kit (11121ES60, Yeasen, Shanghai, China) as per the manufacturer’s instructions. The primer sequences employed in this study are listed in Supplementary Table S2. By analyzing key gene expression differences between the two patient groups and developing a predictive nomogram model, we aimed to achieve differential diagnosis of SS patients with or without comorbid PA.

### 2.9 Immune infiltration analysis by CIBERSORT and scRNA-seq

Utilizing the CIBERSORT algorithm (Newman et al., 2015), we utilized deconvolution on the gene expression matrix that was normalized to ascertain the makeup of infiltrating immune cells present in tissue samples. The algorithm precisely evaluated the relative distribution of 22 diverse types of immune cells. The proportions of these immune cell types across varied groups were quantified with the “CIBERSORT” R package. To illustrate potential correlations among distinct immune cell populations throughout disease progression, we created heatmaps using the “corrplot” R package. Furthermore, single-sample gene set enrichment analysis (ssGSEA) was performed to explore the correlation between immune cell infiltration and the expression patterns of characteristic genes. Visual representations of these associations were generated using the “ggcorrplot” package.

Utilizing the Seurat package (Stuart et al., 2019), we conducted single-cell data analysis, encompassing quality control (QC), dimensionality reduction, and clustering. Cells failing predefined criteria—specifically, those with fewer than 200 detected genes or mitochondrial content exceeding 20%—were removed. The retained high-quality cells underwent linear transformation through the “NormalizeData” and “ScaleData” functions. To address batch effects, we harmonized data from multiple samples with the Harmony package’s “RunHarmony” function (Korsunsky et al., 2019). Cluster distributions were visualized using Uniform Manifold Approximation and Projection (UMAP) (Becht et al., 2018).

### 2.10 Elucidating identified biomarker-hallmark gene set connections by ssGSEA

To investigate potential associations between the identified biomarkers and hallmark gene sets, we utilized the ssGSEA method. The hallmark gene sets are sourced from the Molecular Signatures Database (MSigDB), a comprehensive repository that covers 50 distinct biological states and processe (Liberzon et al., 2011). Employing the Gene Set Variation Analysis (GSVA) tool, we performed ssGSEA to assess the correlations between the potential biomarkers and hallmark gene sets.

### 2.11 Statistical analysis

In our research, SPSS Version 26.0 and GraphPad Prism Version 9.4.0 were employed to perform statistical analyses. The comparison of continuous variables between the two groups was performed using the Student's t-test. *p*-values less than 0.05 was considered statistically significant. The version of R software used was 4.2.1.

## 3 Results

### 3.1 DEGs identification via Limma in SS and PA

**FIGURE 1:**
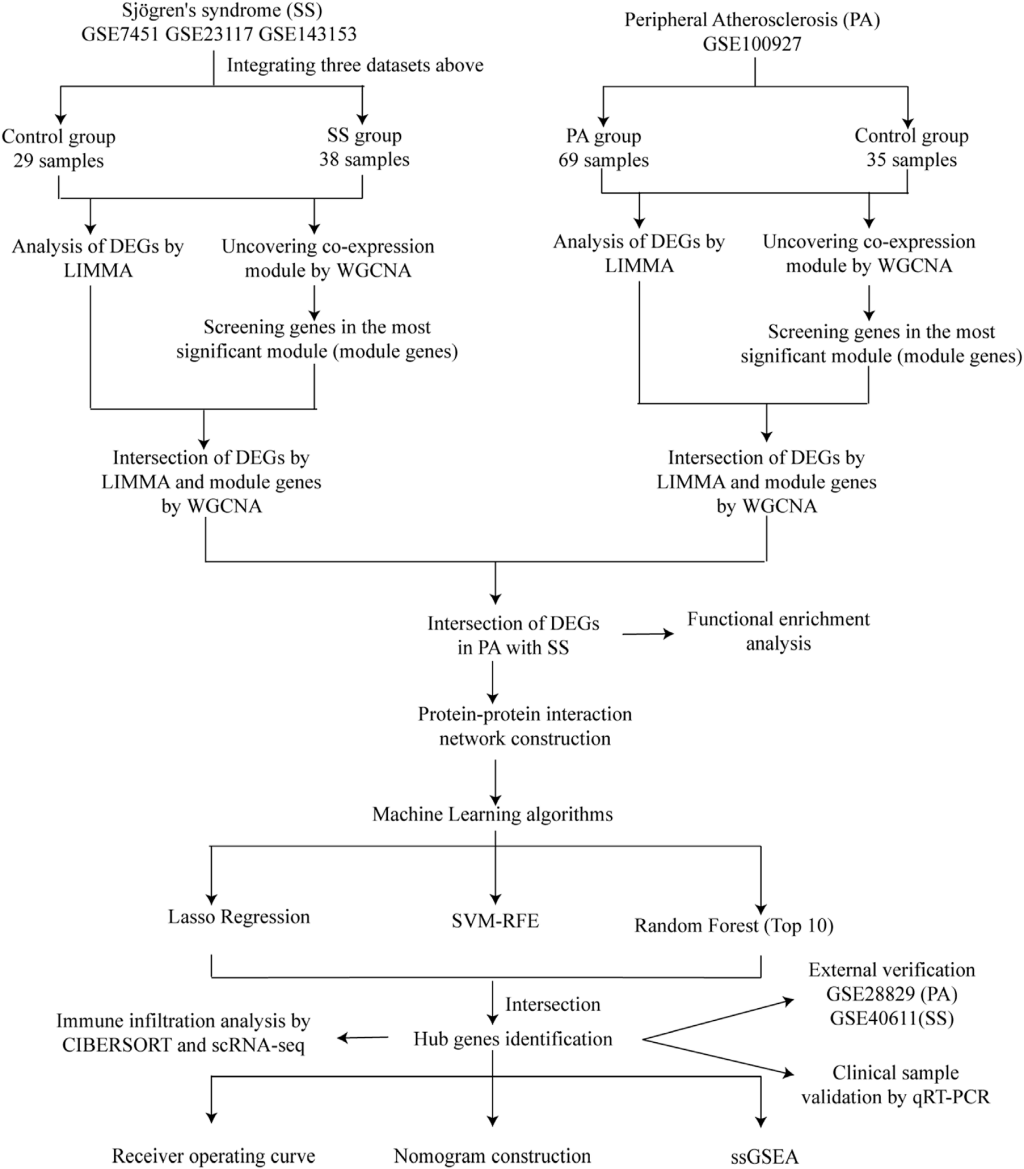
Study flowchart.

**FIGURE 2:**
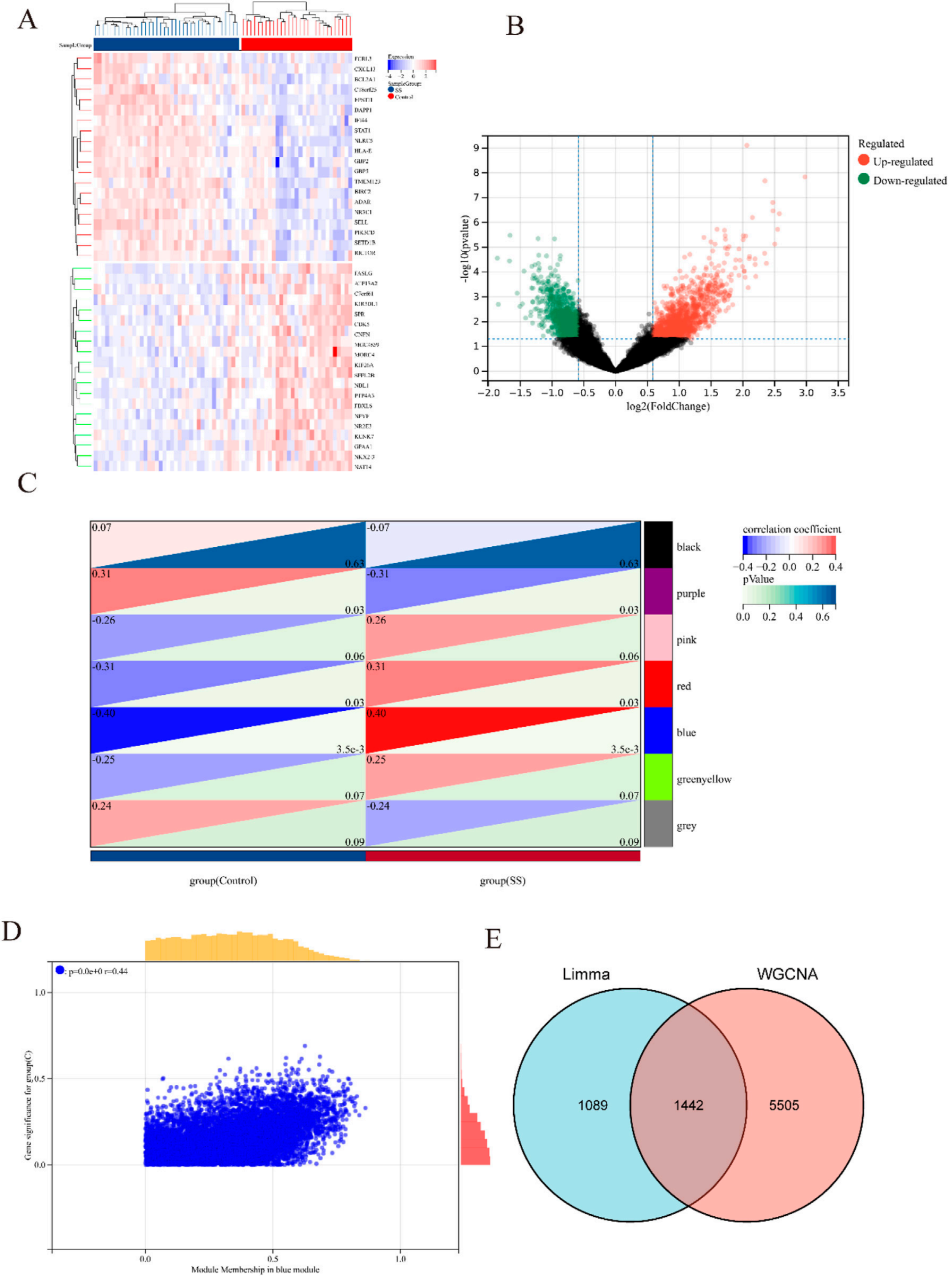
Heatmap and volcano plot of DEGs between SS and control group, and identification of module genes in SS via WGCNA. **(A)** Heatmap showing the top 20 DEGs between SS and control groups, emphasizing the most upregulated and downregulated genes. Blue blocks indicate downregulated expression, while red blocks indicate upregulated expression. **(B)** Volcano plot depicting the DEGs between SS and control groups. Red and green points represent significantly upregulated and downregulated DEGs, respectively. **(C)** Heatmap of SS-related modules. The number in the upper left corner indicates the correlation between the module and SS, and the P value in the lower right corner signifies the significance of this correlation. In SS, the blue module exhibits the strongest correlation. **(D)** Correlation between gene significance and module membership in the blue module. **(E)** Venn diagram illustrating that the intersection of DEGs and significantly expressed module genes in SS results in 1,442 DEGs.

**FIGURE 3:**
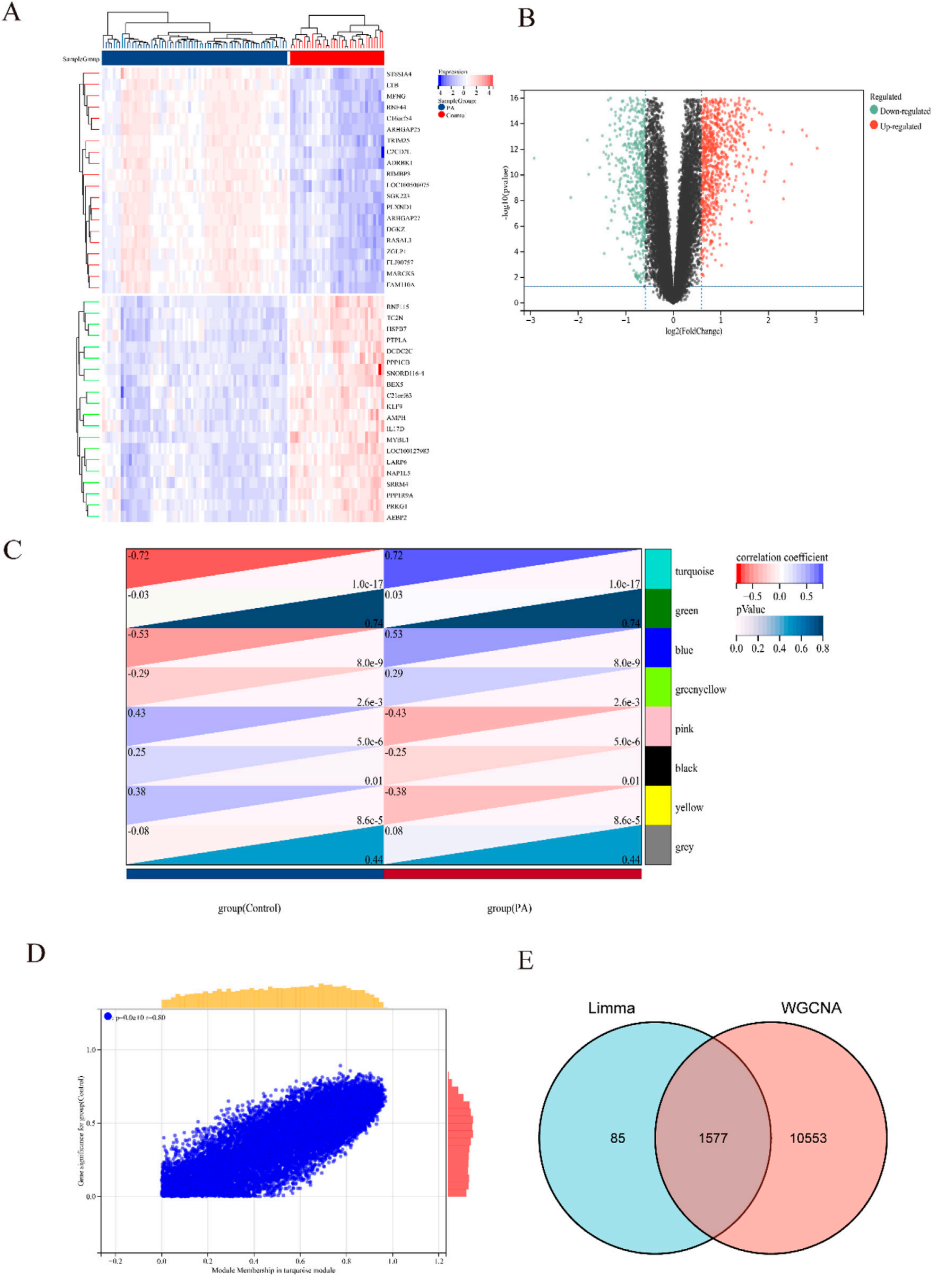
Heatmap and volcano plot of DEGs between PA and control group, and identification of module genes in PA via WGCNA. **(A)** Heatmap showing the top 20 DEGs between PA and control groups, emphasizing the most upregulated and downregulated genes. Blue blocks indicate downregulated expression, while red blocks indicate upregulated expression. **(B)** Volcano plot depicting the DEGs between PA and control groups. Red and green points represent significantly upregulated and downregulated DEGs, respectively. **(C)** Heatmap of PA-related modules. The number in the upper left corner indicates the correlation between the module and PA, and the P value in the lower right corner signifies the significance of this correlation. In PA, the turquoise module exhibits the strongest correlation. **(D)** Correlation between gene significance and module membership in the turquoise module. **(E)** Venn diagram illustrating that the intersection of DEGs and significantly expressed module genes in PA results in 1,577 DEGs.

Figure 1 illustrated the research flowchart. Before batch effect correction, the boxplot (Supplementary Figure S1A) displayed significant diversity in sample distributions among datasets, suggesting the presence of batch effects. Post-correction, data distributions among datasets attained uniformity, aligning medians along a single axis (Supplementary Figure S1B). For PCA, PC1 and PC2 were plotted to visualize the first two principal components. Before batch correction, PC1 and PC2 explained >70% of variance, with dataset-specific differences dominating, reflecting strong batch effects (Supplementary Figure S1C). After correction, samples from different datasets interclustered and intertwined, demonstrating batch effect elimination (Supplementary Figure S1D). 2,531 DEGs were identified in total. Among them, 1,392 genes showed upregulation, while 1,139 genes exhibited downregulation in the SS group. In comparison to the control group, the PA group revealed 1,662 DEGs, with 1,055 genes upregulated and 607 genes downregulated. We visualized the top 20 upregulated and downregulated DEGs using a heatmap and presented the overall distribution of all identified DEGs through a volcano plot. In the volcano plot, the log2 fold change (log2FC) of gene expression is displayed on the x-axis, whereas the -log10(P-value), which reflects the statistical significance of differential gene expression, is plotted on the y-axis. Each gene is represented as a point: upregulated genes are shown in red, downregulated genes in blue, and genes without significant differential expression in black (SS: Figures 2A,B, PA; Figures 3A,B).

### 3.2 Identification of significant module genes in SS and PA using WGCNA

Respective significant module genes associated with SS and PA were identified using WGCNA. In Figure 2C, the blue module exhibited a significant positive correlation with SS (r = 0.4, p = 3.5 × 10^−3^), and the turquoise module showed a strong positive correlation with PA (r = 0.72, p = 1.0 × 10^−17^) in Figure 3C. The relationship between module membership in the blue/turquoise modules and gene significance in SS and PA is illustrated in Figures 2D, 3D. Supplementary Figures S2, S3 shows the soft threshold selection and gene clustering trees. Specifically, 6,947 genes were selected for SS and 12,130 for PA. The overlap of 2531 SS-related DEGs and 1,342 module genes associated with SS yielded 1442 SS-related DEGs (Figure 2E). Similarly, the overlap of 1,662 DEGs related to PA and 12,130 module genes associated with PA resulted in 1577 PA-related DEGs (Figure 3E).

### 3.3 Functional enrichment analysis of SS-related DEGs in PA

**FIGURE 4:**
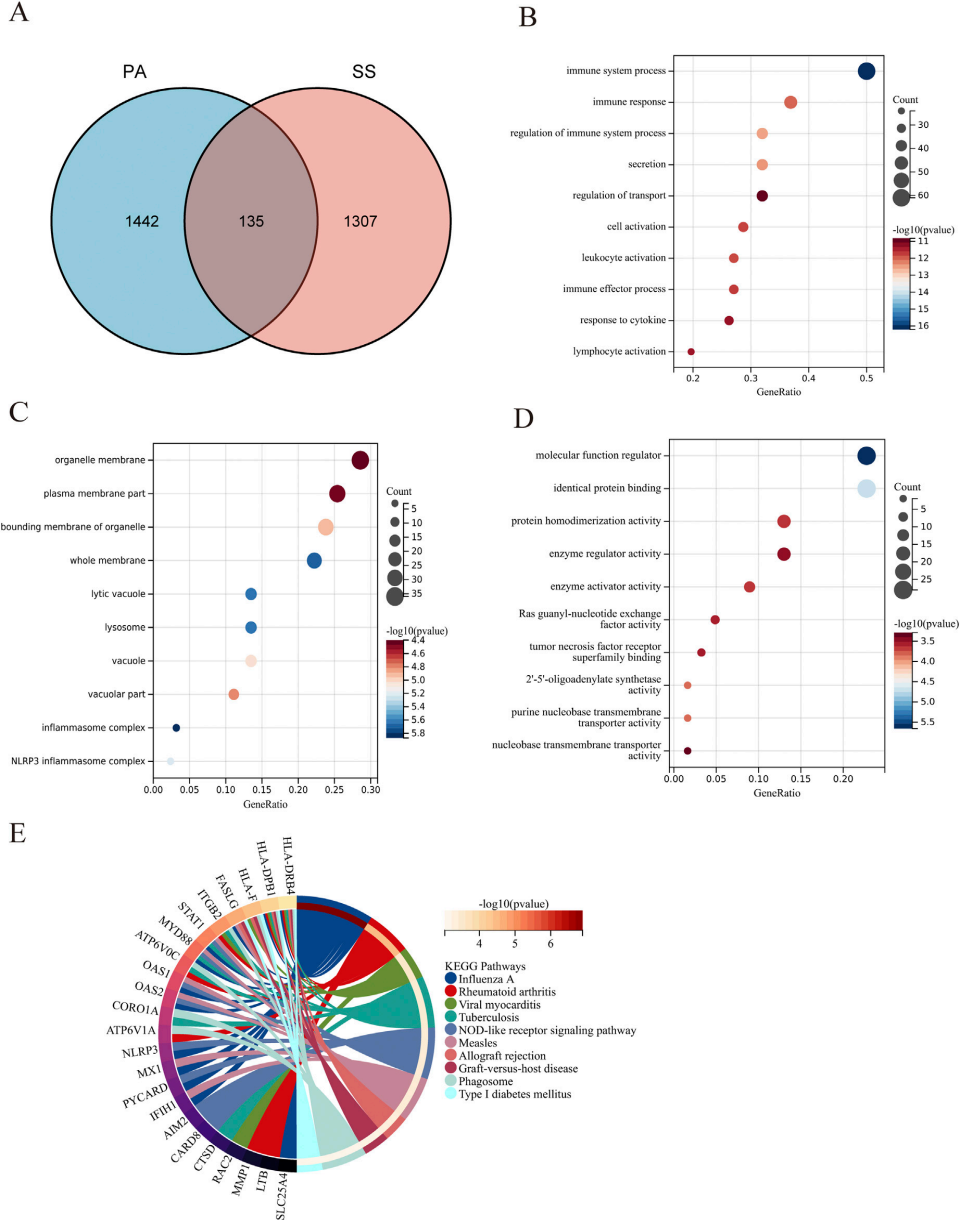
Functional enrichment analysis of DEGs associated with SS in PA. **(A)** The Venn diagram illustrates that the overlap of DEGs between PA and SS resulted in 135 SS-related differentially expressed genes in PA. **(B–D)** GO analysis of DEGs associated with SS in PA, covering biological process, cellular component, and molecular function. The X-axis indicates the gene ratio, while the Y-axis denotes various ontologies. The size and color of the circles reflect the number and significance of the genes. **(E)** KEGG pathway analysis highlights the primary signaling pathways implicated in SS-related DEGs in PA.

The overlap of 1,442 DEGs associated with SS and 1,577 DEGs linked to PA produced 135 DEGs connected to both SS and PA (Figure 4A). The significant enrichment of GO terms in biological processes (BP) for these 135 DEGs encompassed “immune system process,” “immune response,” and “regulation of immune system process.” Furthermore, the enriched terms for CC include “organelle membrane,” “plasmamembrane part,” and “bounding membrane of organelle,” while the MF of DEGs were closely tied to “molecular function regulator,” “identical protein binding,” and “protein homodimerization activity” (Figures 4B–D; Supplementary Table S3). The outcomes of the functional enrichment analysis for these 135 DEGs were presented in Figure 4E and Supplementary Table S1. These DEGs exhibited notable enrichment in “influenza A,” “rheumatoid arthritis,” and “viral myocarditis.”

### 3.4 PPI network construction and hub gene selection

**FIGURE 5:**
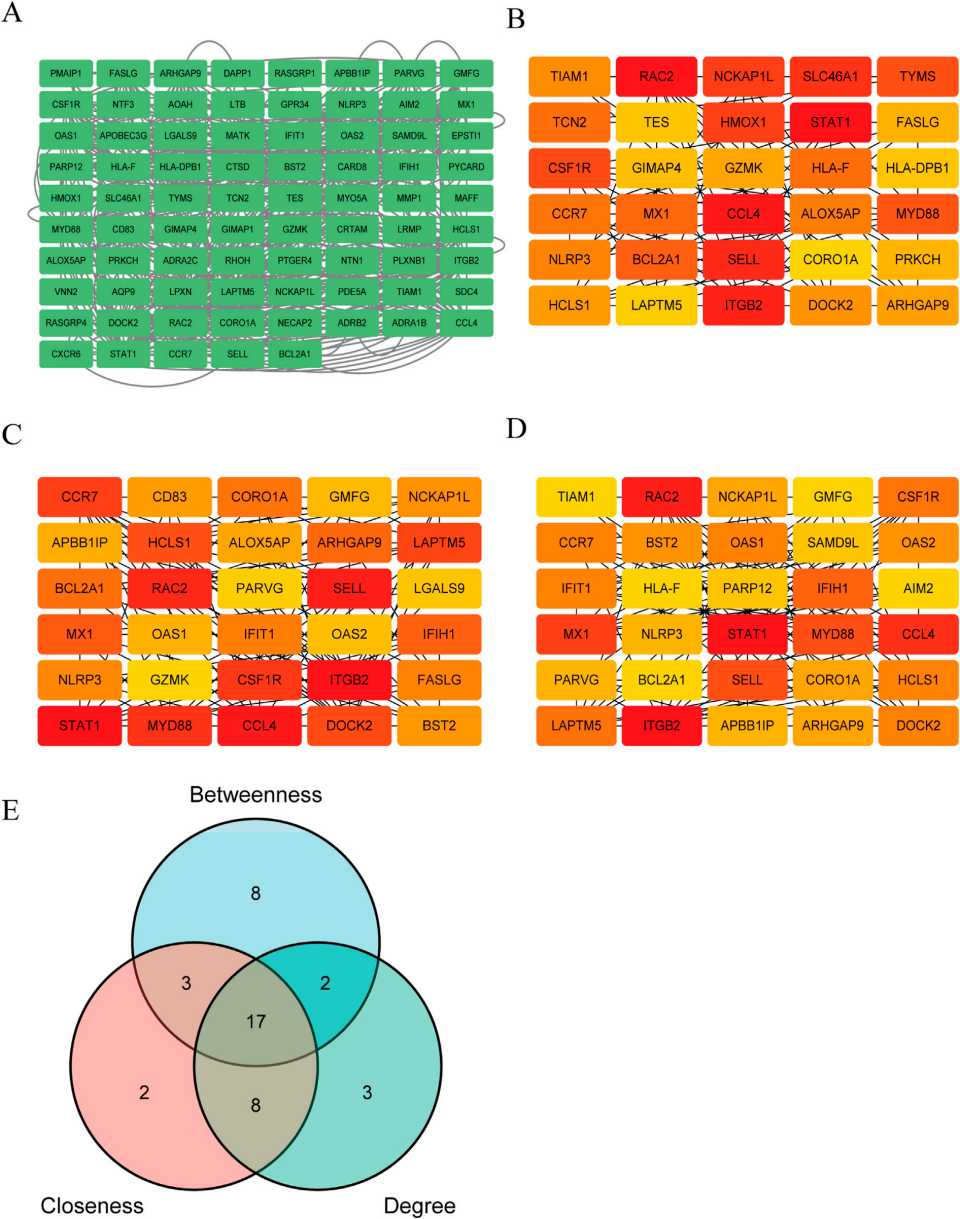
Construction of PPI network and selection of key Genes. **(A)** The PPI network of 77 PA and SS-related DEGs was visualized using Cytoscape software. Due to the lack of interactions among some genes, 58 DEGs were excluded from the network, resulting in a PPI network consisting of 77 nodes (representing genes) and multiple edges (indicating gene interactions). **(B–D)** The CytoHubba plugin in Cytoscape was employed to identify key genes from the 77 genes using three distinct algorithms. By analyzing these genes from three different perspectives, the top 30 genes were selected for each algorithm. **(B–D)** depict the Betweenness, Closeness and Degree algorithms, respectively. Deeper colors indicate a more significant role in the algorithm. **(E)** The intersection of the results from the three algorithms was determined, and ultimately, 17 DEGs were selected for further in-depth analysis.

135 DEGs was used to construct a PPI network. Using Cytoscape, the comprehensive PPI network comprising 77 DEGs associated with both SS and PA was visualized (Figure 5A). 58 DEGs were excluded due to a lack of interactions. Furthermore, three distinct algorithms from the Cytoscape plug-in CytoHubba were utilized to identify overlapping DEGs. Figures 5B–D displayed the top 30 DEGs derived from the intersection of these algorithms. For subsequent machine learning analysis, 17 DEGs were chosen based on a Venn diagram (Figure 5E; Supplementary Table S4).

### 3.5 Identification of candidate diagnostic genes using machine learning algorithms

**FIGURE 6:**
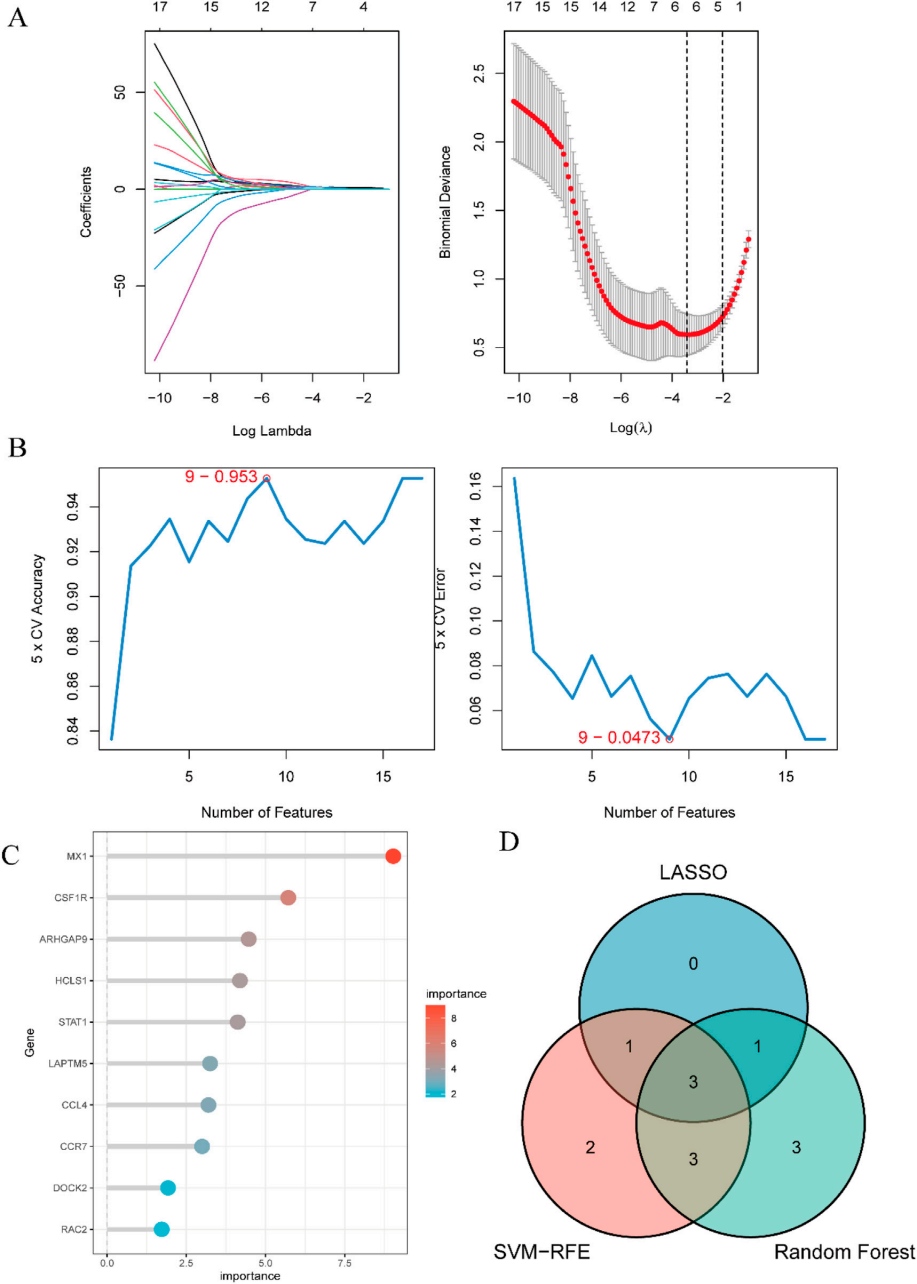
Identification of candidate predictive biomarkers using machine learning algorithms. **(A)** Lasso regression analysis was performed to screen a series of gene variables, using binomial deviation as an evaluation metric, and identified 5 genes with the lowest binomial deviation. **(B)** The SVM-RFE algorithm was applied to minimize error and maximize accuracy by iteratively eliminating less important genes from the gene set, ultimately selecting 9 genes with the lowest error and highest accuracy. **(C)** The random forest algorithm ranked genes based on their importance scores, resulting in the selection of the top 10 genes. **(D)** A venn diagram was used to visually illustrate the intersection of the three machine learning algorithms, identifying 3 hub genes: CCL4, CSF1R, and MX1.

The outcome of the Lasso regression analysis was illustrated in Figure 6A, revealing that 5 DEGs exhibited the least binomial variance. Through the utilization of Support Vector Machine-Recursive Feature Elimination (SVM-RFE), the top 9 DEGs with the highest precision and lowest error rates were selected (Figure 6B). The random forest algorithm was employed to assess the significance of the DEGs, as depicted in Figure 6C, showcasing the top 10 DEGs based on their importance ranking. Subsequently, three crucial DEGs (CCL4, CSF1R, MX1) chosen from the intersecting region in the Venn diagram were identified for ROC evaluation (Figure 6D).

### 3.6 The predictive value evaluation, nomogram construction and validation of the hub genes

**FIGURE 7:**
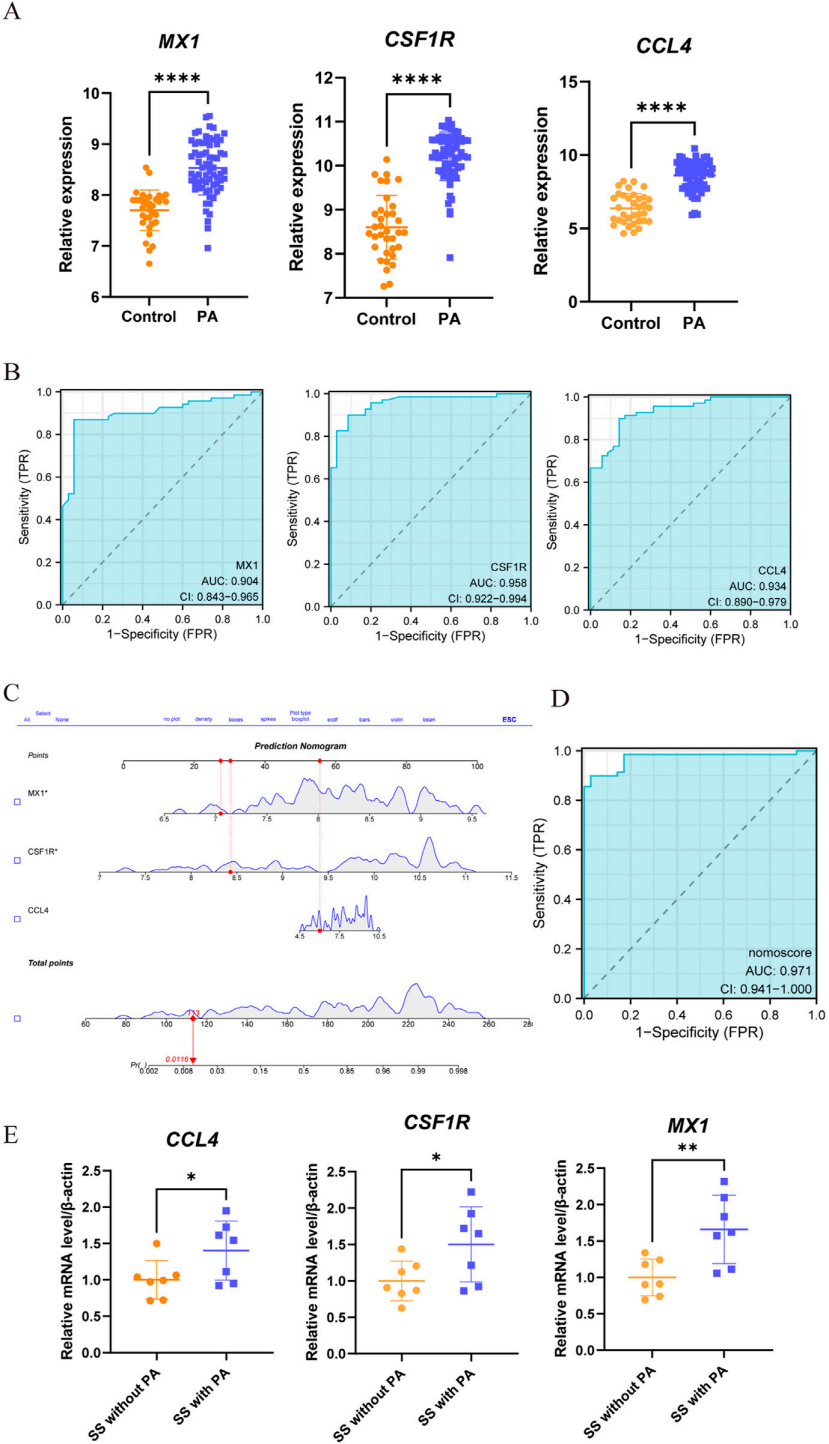
Assessment of the predictive value and construction of nomogram for candidate biomarkers in PA. **(A)** Significant differences in the expression levels of three candidate genes between PA patients and controls, with increased expression (****, P < 0.0001). **(B)** ROC curve analysis was conducted to assess the predictive value of these three genes for PA. Each panel clearly displays the area under the curve (AUC) value and its corresponding 95% confidence interval. A higher AUC value indicates that the diagnostic model has better discriminatory power and can more accurately distinguish PA patients from healthy controls. **(C,D)** A nomogram, a visual predictive model that integrates multiple predictive factors (CCL4, CSF1R, and MX1), was constructed for PA. Panels C and D show the process and results of constructing the nomogram based on these three genes. **(E)** Through qRT-PCR, differential expression of three genes was detected when comparing the SS patients with PA against those without PA (*, p < 0.05; **, p < 0.01).

The comparison with the control group revealed upregulation of three genes (CCL4, CSF1R, MX1) in PA (Figure 7A). The ROC curve analysis indicated good diagnostic performance for each gene (Figure 7B): MX1 (AUC: 0.904, 95% CI 0.843–0.965); CCL4 (AUC: 0.934, 95% CI: 0.890–0.979); CSF1R (AUC: 0.958, 95% CI 0.922–0.994). Subsequently, the nomogram was generated (Figure 7C). ROC analysis of the nomogram assessed its clinical utility, demonstrating high predictive value for PA (AUC 0.970, 95% CI 0.941–1.000) (Figure 7D). To validate its predictive potential, we utilized the GSE28829 validation dataset for ROC curve analysis. As illustrated in Supplementary Figure S4A, the genes CCL4, CSF1R, and MX1 exhibited upregulation. Supplementary Figure S4B presents the AUC and 95% CI for each gene. And ROC analysis demoonstrated satisfactory predictive capabilities for these genes. Lastly, the nomogram construction is depicted in Supplementary Figure S4C. The evaluation of the nomogram in the validation dataset yielded an AUC of 0.952, indicating significant clinical predictive ability, as shown in Supplementary Figure S4D.

SS also demonstrated elevated expression of genes (CCL4, CSF1R, MX1), as depicted in Supplementary Figure S5A. The nomogram, which is presented in Supplementary Figure S5B, achieved an AUC of 0.724 (95% CI: 0.600–0.848). This outcome, as illustrated in Supplementary Figure S5C, validates its clinical applicability in diagnosis. To evaluate its predictive capacity, we utilized the GSE40611 validation dataset for ROC curve analysis. Supplementary Figure S5D illustrates the construction of the nomogram. In the validation group, the nomogram demonstrated an AUC of 0.961, clinical its significant clinical predictive capacity, as depicted in Supplementary Figure S5E.

Moreover, as depicted in Figure 7E, clinical verification of peripheral blood samples revealed elevated expression of three genes (CCL4, CSF1R, MX1) in SS patients with PA compared to the SS-only group. Additionally, we developed a nomogram to evaluate the risk of PA in SS patients. Demonstrated in Supplementary Figures S6A, B, this model achieved an AUC of 1, confirming its remarkable predictive capability for PA in SS patients.

### 3.7 Immune cell infiltration analysis

**FIGURE 8:**
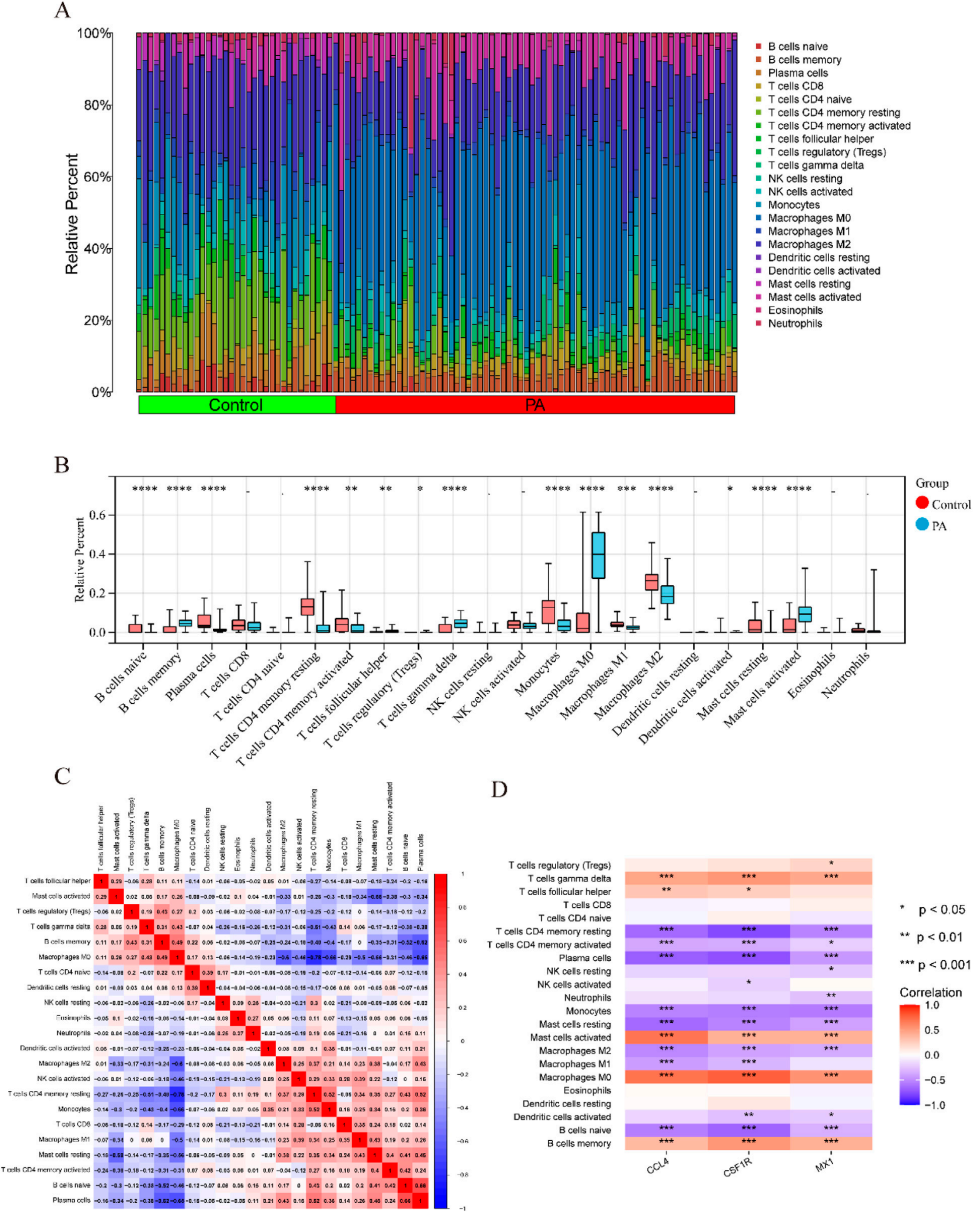
Comparison of immunological changes between the control group and the PA group, along with the association between three key hub DEGs and immune-related characteristics in PA. **(A)** A bar plot visually illustrates the relative abundances of 22 different immune cell types across all samples. **(B)** A boxplot illustrates the intergroup differences in immune cell expression levels between the PA and control groups (*p < 0.05, **p < 0.01, ***p < 0.001, ****p < 0.0001). **(C)** A heatmap illustrates the relationships between different immune cell types. Rows and columns correspond to distinct immune cell types, with color intensity denoting correlation strength. Red signifies a positive correlation and blue denotes a negative correlation. **(D)** A correlation analysis diagram evaluates the link between immune cell infiltration and the three hub DEGs. Similar to the prior depiction, red signifies a positive correlation and blue denotes a negative correlation, with color intensity denoting correlation strength.

In PA,immune cell infiltration analysis using the CIBERSORT algorithm revealed the distribution of 22 different types of immune cells in each sample (Figure 8A). Boxplot analysis demonstrated that, compared to the control group, the proportions of γδ T cells, memory B cells, activated mast cells, and M0 macrophages were significantly higher in the PA samples. Conversely, the levels of naive B cells, plasma cells, CD4 memory resting T cells, CD4 memory activated T cells, monocytes, M1 macrophages, M2 macrophages, and resting mast cells were significantly lower in the PA samples (Figure 8B). Further correlation analysis revealed a positive correlation between naive B cells and plasma cells (r = 0.66) and a significant negative correlation between M0 macrophages and CD4 memory resting T cells (r = −0.78) (Figure 8C). Additionally, the immune cell infiltration analysis showed a significant correlation with three hub DEGs (Figure 8D). In SS, Supplementary Figure S7A showed the distribution of 22 immune cell types per sample. Boxplot analysis revealed higher proportions of follicular helper T cells and neutrophils in SS samples compared to controls, while monocytes were significantly reduced (Supplementary Figure S7B). Correlation analysis identified a positive correlation between monocytes and resting NK cells (r = 0.54) and a significant negative correlation between γδ T cells and CD8 T cells (r = −0.48) (Supplementary Figure S7C).

**FIGURE 9:**
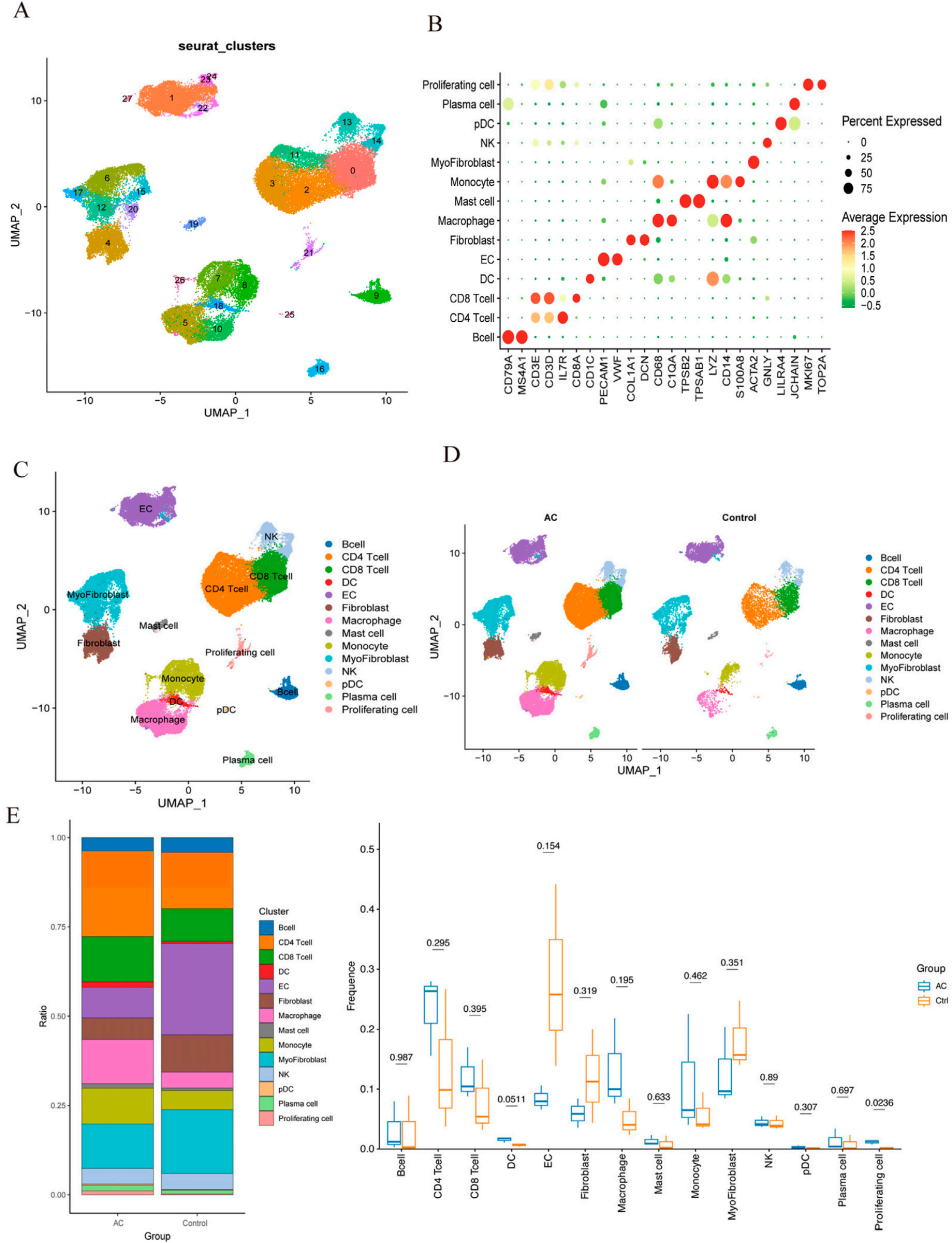
Single-cell RNA sequencing of human atherosclerotic plaque tissues. **(A)** The single-cell atlas of carotid atherosclerotic plaques was visualized through UMAP. **(B)** Dot plot illustrating the proportion of cells expressing specific genes (dot size) and the mean expression levels in expressing cells (dot color) across distinct clusters. **(C)** This representation delineated 14 distinct cell types. **(D)** An overview comparing the 14 cell types between the AC and Control groups was conducted and categorized by cell type. **(E)** The proportions of cell types in each group were compared using bar charts and box plots.

Following quality control (QC), we conducted normalization, unsupervised dimensionality reduction, and UMAP clustering analysis. This analysis yielded 28 major cell clusters (Figure 9A). Using marker genes of various cell types, we annotated the data, categorizing the 28 clusters into 14 cell types and displaying marker genes for each subgroup (Figures 9B,C). Figures 9D,E showed that endothelial cells (EC), myofibroblasts, and CD4 T cells comprised the top three cell clusters in the PA group, whereas CD4 T cells, CD8 T cells, and myofibroblasts dominated the AC group. Although not statistically significant, boxplots showed that PA samples had higher proportions of EC, fibroblasts, and myofibroblasts compared to the AC group. Conversely, PA samples exhibited lower proportions of CD4 T cells, CD8 T cells, macrophages, and monocytes.

In summary, the immune cell regulation mechanism may represent a promising therapeutic approach for SS-related PA.

### 3.8 ssGSEA

Using the ssGSEA method, we conducted an in-depth investigation into the relationships between the three hub genes and various biological processes. The results demonstrated that these core genes were significantly associated with multiple biological processes. The biological processes positively correlated with these three genes included “inflammatory response,” “IL6/JAK/STAT3 signaling,” “coagulation,” “apoptosis,” and “angiogenesis,” etc., while the negatively correlated ones were “Hedgehog signaling,” “myogenesis,” and “TGF-β signaling,” etc., as shown in Supplementary Figure S8. These findings provide valuable insights into the pathophysiological mechanisms underlying SS-induced PA and guide future research and the identification of potential therapeutic targets.

## 4 Discussion

Sjögren’s syndrome (SS) is a complex chronic autoimmune disease that primarily affects the blood system and organs such as the lungs and kidneys (Gao et al., 2020). It shares some common pathophysiological features with systemic lupus erythematosus and can also impact blood vessels. Recent reports have highlighted a close association between SS and the development of atherosclerosis, leading to an elevated risk of cardiovascular and cerebrovascular events. Atherosclerosis (AS) is an inflammatory condition of the arteries and a significant contributor to peripheral vascular disease, potentially causing vascular stenosis or occlusion, resulting in tissue and organ ischemia (Yi et al., 2022). Although its progression is often subtle, it poses a severe threat. An expanding body of research indicates that peripheral atherosclerosis (PA) is an inflammatory disorder closely linked to autoimmune conditions. Acute systemic inflammatory responses and chronic systemic vasculitis contribute to endothelial dysfunction, fostering the formation of atherosclerotic plaques, which can subsequently give rise to cardiovascular and peripheral vascular disorders (Frostegård, 2005). Although there is a scarcity of research data on PA in patients with SS, the harm of PA in this patient population should not be overlooked. Therefore, early prevention, diagnosis, and treatment of PA in patients with SS are of paramount importance.

To tackle this issue, we employed comprehensive bioinformatics and machine learning technologies for an exhaustive analysis of microarray datasets, with the goal of identifying common biomarkers and biological pathways linked to these two diseases. These methodologies offer a robust means of accurately pinpointing disease-related biomarkers, thereby facilitating research into comprehending disease onset, advancement, and potential pathogenic mechanisms. In this study, we utilized the Limma R package, a potent tool for gene expression data analysis, and WGCNA, an analytical instrument adept at discerning significant correlations between gene modules and phenotypes, to pinpoint DEGs associated with SS and PA. Through overlap analysis, a final compilation of 135 DEGs closely intertwined with SS and PA emerged. To shed light on the functional roles of these 135 DEGs, we conducted enrichment analysis using the KEGG pathway and GO databases. The outcomes underscored their primary associations with “immune system process,” “immune response,” and “regulation of immune system process,” all closely related to the occurrence of SS and PA, reinforcing the hypothesis of a possible connection between the two conditions. Additionally, a PPI network was established using the 135 DEGs to explore potential interactions, revealing 17 potential hub DEGs that might significantly influence underlying biological processes. Machine learning algorithms, such as SVM-RFE, LASSO, and random forests, enable the extraction of valuable insights from complex biological data, with their amalgamation enhancing the precision and dependability of biomarker prognostication. This study employed three machine learning techniques and ROC curve analysis to evaluate the diagnostic accuracy of candidate gene expressions, culminating in the identification of three candidate hub genes (CCL4, CSF1R, and MX1) with promising diagnostic utility. Construction of a Nomogram using these gene sets showcased a high predictive value for PA in SS. To validate the prognostic efficacy of these genes, an additional dataset (GSE28829) was employed for validation, affirming their practical application value.

CCL4 (C-C motif chemokine ligand 4), commonly referred to as MIP-1β (Macrophage Inflammatory Protein-1 beta), is an important chemokine within the CC chemokine family. It is primarily synthesized by immune cells such as monocytes, macrophages, and T cells, playing a crucial role in the modulation of immune responses and inflammatory processes. By binding to its receptor CCR5, CCL4 facilitates the migration and activation of immune cells, thereby exerting a substantial influence in a variety of immune-related conditions. In SS, the expression of CCL4 is closely associated with the disease’s pathogenesis. Studies indicate that CCL4 is significantly elevated in the salivary glands and peripheral blood of SS individuals, potentially facilitating lymphocyte infiltration and local inflammatory responses (Blokland et al., 2021). The hallmark histological feature of SS is the infiltration of mononuclear cells into exocrine glands, with the secretion of CCL4 potentially playing a pivotal role in the immunopathology of SS (Lee et al., 2010; Mircheff et al., 2019). In the context of atherosclerosis, CCL4 also plays a significant role. Atherosclerosis is characterized as a chronic inflammatory condition, with CCL4 detectable in T cells, smooth muscle cells, and macrophages within atherosclerotic plaques, and further upregulated in vulnerable plaques. CCL4 contributes to the formation and progression of atherosclerotic plaques by promoting the migration of monocytes and T cells (Chang et al., 2020). Research has shown that chemokines play a crucial role in the advancement of cardiovascular diseases (Gencer et al., 2021). Elevated levels of CCL4 in the plasma of patients with atherosclerosis indicate a higher risk of stroke and cardiovascular events for those with higher CCL4 levels (Tatara et al., 2009). Therefore, CCL4 not only plays a significant role in Sjögren’s syndrome but also has a key function in the pathophysiological process of atherosclerosis. In summary, as an important chemokine, CCL4 demonstrates significant pathological relevance in both Sjögren’s syndrome and atherosclerosis, indicating its potential as a therapeutic target for these two diseases.

CSF1R (Colony Stimulating Factor 1 Receptor) is a coding gene that encodes a tyrosine kinase receptor. It is predominantly expressed in monocytes and macrophages, playing a pivotal role in immune response modulation, cell proliferation, and differentiation (Sletta et al., 2021). Recent studies have identified mutations in CSF1R linked to inflammation and immune disorders, including Sjögren’s syndrome and atherosclerosis. Evidence suggests that CSF1R significantly contributes to the pathogenesis of Sjögren’s syndrome, with mutations or aberrant expression potentially inducing macrophage dysfunction, intensifying inflammatory responses, and promoting tissue damage (Hu et al., 2022). The second ligand of CSF1R, IL-34, is a recently discovered inflammatory cytokine, has associations with various rheumatic conditions such as rheumatoid arthritis, systemic lupus erythematosus, and SS. Elevated IL-34 expression in the salivary glands of SS patients is believed to be implicated in the disease’s pathogenesis (Ciccia et al., 2013; Liu et al., 2020). These findings propose that CSF1R may function as a promising biomarker and therapeutic target for SS. Furthermore, CSF1R plays a crucial role in atherosclerosis pathogenesis. Study demonstrates that CSF1R activation promotes macrophage proliferation and differentiation, pivotal in atherosclerotic plaque formation and progression. Inhibiting CSF1R may aid in attenuating atherosclerosis advancement (Wei et al., 2015). Bioinformatics analyse also support the significant involvement of CSF1R in atherosclerosis biological processes (Teng et al., 2023). CSF1R may play an important role in the pathogenesis of SS and PA, suggesting its potential as a diagnostic and therapeutic target warranting further exploration.

MX1 (MX Dynamin Like GTPase 1) gene encodes a protein that is a member of the interferon-induced Mx protein family. Study has shown that the expression of the IFN-stimulated gene MX1 is elevated in patients with SS (Jara et al., 2021). In SS patients, the expression levels of MX1 in the glands and peripheral blood are increased, and utilizing a random forest model with the MX1 gene expression level as a significant feature shows promise as a diagnostic technique for SS (Xu et al., 2023). Atherosclerosis is a chronic inflammatory disease, and research has found that the MX1 gene may also play a role in the occurrence and development of atherosclerosis. The antiviral function of MX1 is closely related to its role in regulating inflammatory responses, potentially affecting the pathological processes of atherosclerosis. MX1 may be a potential biomarker for atherosclerosis (Wang et al., 2024). In patients with systemic lupus erythematosus, overexpression of MX1 may be associated with accelerated atherosclerosis. The expression of MX1 may lead to endothelial cell damage by regulating the release of inflammatory factors and the activation of immune cells, ultimately affecting the progression of atherosclerosis (Lee et al., 2007). MX1 may play an important role in both SS and PA; However, the precise relationship between SS and PA remains to be elucidated.

The immune infiltration analysis in this study revealed that in patients with SS, the increased abundance of follicular helper T cells (Tfh) and neutrophils, likely mediated by the activation of the Tfh-B cell axis and the release of neutrophil extracellular traps (NETs) (Carmona-Rivera et al., 2015), leads to vascular endothelial damage and the onset of inflammatory cascades. Previous studies have shown that Tfh cells induce B cells to differentiate into plasma cells via IL-21 secretion (notably, a significant positive correlation was observed between naive B cells and plasma cells in this study, r = 0.66), leading to the production of anti-endothelial cell antibodies that directly harm the vascular endothelium (Dale et al., 2019; Ricard et al., 2019). Additionally, research has indicated that the release of NETs by neutrophils can worsen vascular endothelial injury by generating cytotoxic proteases (e.g., histones, elastase, myeloperoxidase MPO), and pro-inflammatory mediators (Wang et al., 2021), ultimately contributing to the development of atherosclerosis (Clement et al., 2015). In PA samples, the concurrent increase of γδ T cells and M0 macrophages further exacerbates the imbalance of innate immune responses. Studies have shown that γδ T cells stimulate neutrophil mobilization and activation by releasing IL-17, thereby promoting the production of pro-inflammatory cytokines and chemokines (Caccamo et al., 2011). Meanwhile, another study (Yin et al., 2022) has indicated that due to polarization irregularities (evidenced by a significant decrease in M1/M2 macrophage numbers), M0 macrophages provoke an imbalance between pro-inflammatory and anti-inflammatory responses, worsening vascular endothelial injury and initiating atherosclerosis.

Single-cell transcriptome analysis delineated the distribution patterns of immune cells during atherosclerosis progression. The findings highlighted that CD4 T cells and CD8 T cells constituted the primary components of the immune cell population in the atherosclerotic core region (AC group). Comparatively, the AC group exhibited significantly increased proportions of CD4 T cells, CD8 T cells, macrophages, and monocytes in contrast to the control group, emphasizing the substantial role of immune cells in the advancement of atherosclerotic plaques.

Inflammation may accelerate atherosclerosis by promoting inflammatory cell infiltration into the vascular wall and causing endothelial dysfunction. Even in patients with SS lacking cardiovascular disease or risk factors, endothelial dysfunction and impaired endothelial function may persist, suggesting a predisposition to atherosclerosis (Łuczak et al., 2021). Therefore, immune modulation may be an option for PA in SS patients.

Furthermore, ssGSEA analysis identified significant associations between the three hub genes and various biological processes. The positive correlation between hub genes and inflammatory responses, as well as the IL6/JAK/STAT3 signaling pathway, indicates that there is persistent immune activation and a pro-inflammatory environment during the pathogenesis of SS-PA. SS is a chronic systemic inflammatory disease, and PA is an inflammatory response secondary to vascular injury. Inflammatory responses continuously affect the entire process of arteriosclerosis (Li et al., 2018). Meanwhile, the abnormal activation of the IL6/JAK/STAT3 pathway can upregulate VCAM-1, accelerate endothelial cell damage, and promote arteriosclerosis (Wiejak et al., 2019). Inflammation and angiogenesis are often interrelated, and the imbalance of angiogenesis can lead to various inflammatory diseases such as SS (Lisi et al., 2013), and its abnormal activation is also closely related to the occurrence of PA (Lu et al., 2011). It is worth noting that hub genes are positively correlated with apoptosis: study has shown that apoptosis plays a core role in the pathogenesis of SS (Sisto et al., 2006), and apoptotic cells are specifically present in the calcified regions of arteries in patients with atherosclerosis (Schaub et al., 2019). In contrast, hub genes are negatively correlated with Hedgehog, myogenesis, and TGF-β signaling pathways, further confirming the defect in vascular repair ability in SS-PA: Hedgehog signaling can regulate angiogenesis (Doboszewska et al., 2023), and its inhibition may promote the occurrence of atherosclerosis; the inhibition of myogenesis may weaken the differentiation potential of vascular smooth muscle cells (VSMCs), leading to the inability of damaged endothelial cells and vascular walls to be effectively repaired, accelerating the progression of atherosclerosis (Doboszewska et al., 2023; Ackers-Johnson et al., 2015). TGF-β may play a core role in both normal and pathological vascular repair, and its functional inhibition may promote the occurrence of PA (Toma and McCaffrey, 2012). These processes are likely to play crucial roles in the occurrence and development of PA in SS patients, suggesting a strong correlation between the two conditions. These findings provide valuable insights into the pathophysiological mechanisms underlying SS-induced PA and guide future research and the identification of potential therapeutic targets.

Our study integrated bioinformatics and machine learning methodologies to identify biomarkers associated with PA induced by SS. The nomogram model developed in our research demonstrated significant predictive capability for PA in SS patients. Furthermore, our study revealed potential disease mechanisms and offers various avenues for investigating the molecular mechanisms of SS-induced PA in future research. Despite these strengths, the study does have limitations. While validation datasets and clinical samples were utilized to evaluate predictive performance, additional experimental investigations are essential to validate and delve into the mechanisms underlying PA induced by SS.

## 5 Conclusion

This study systematically identified three promising hub genes (CCL4, CSF1R, and MX1) and developed a nomogram for predicting PA in SS. Analysis of immune cell infiltration demonstrated that dysregulated immune cells significantly contribute to the progression of PA. Additionally, ssGSEA analysis offered important insights into the mechanisms by which SS leads to PA.

## Data Availability

The original contributions presented in the study are included in the article/Supplementary Material, further inquiries can be directed to the corresponding author.
