# Supplementary material for: Exploration of potential biomarkers and immune cell infiltration characteristics for peripheral atherosclerosis in sjögren’s syndrome based on comprehensive bioinformatics analysis and machine learning
Source: Front Genet. 2025 Jul 30;16:1546315. doi: 10.3389/fgene.2025.1546315 (PMC12343227; doi:10.3389/fgene.2025.1546315)
Supplement: Supplementary file 1 [file DataSheet1.docx]

**Supplementary Material**

**Supplementary Table S1.** Clinical characteristics of the patients.

| Characteristics | SS without PA | SS with PA | *P* value |
| --- | --- | --- | --- |
|  | N=7 | N=7 |  |
| Age, years | 55±10.44 | 65.43±7.35 | 0.052 |
| Gender, n (%) |  |  | 1 |
| Male | 1(14.29) | 2(28.57) |  |
| Female | 6(85.71) | 5(71.43) |  |
| hs-CRP, mg/L | 1.37±1.31 | 0.51±0.21 | 0.13 |
| ESR, mm/h | 26.71±11.22 | 28.86±16.55 | 0.78 |
| BMI, kg/m^2^ | 24.24±3.95 | 21.92±2.36 | 0.21 |
| HbA1c, % | 5.23±0.44 | 5.89±0.71 | 0.059 |
| Diabetes, n (%) | 0(0) | 2(28.57) | 0.46 |
| Hypertension, n (%) | 2(28.57) | 4(57.14) | 0.59 |
| Smoking, n (%) | 1(14.29) | 1(14.29) | 1 |
| Hyperlipidemia, n (%) | 2(28.57) | 2(28.57) | 1 |

Abbreviations: hs-CRP, hypersensitive C-reactive protein; ESR, Erythrocyte Sedimentation Rate; BIM, Body Mass Index; HbA1c, Glycated Haemoglobin A1c.

**Supplementary Table S2.** Primers used in the study.

| Gene | Forward primer | Reverse primer |
| --- | --- | --- |
| CCL4 | AAGCTCTGCGTGACTGTCCTGT | AAGCTTCCTCGCGGTGTAAGA |
| CSF1R | TGAGCAAGACCTGGACAAGGA | CCATTGGTCAACAGCACGTTA |
| MX1 | CAATCAGCCTGCTGACATTG | TGTCTCCTGCCTCTGGATG |
| GAPDH | CGGAGTCAACGGATTTGGTCGT  AT | AGCCTTCTCCATGGTGGTGAAGAC |

**Supplementary Table S3.** Functional enrichment analysis of SS-related DEGs in PA.

| ID | Description | Count | Gene |
| --- | --- | --- | --- |
| **Biological Process** | |  |  |
| GO:0002376 | immune system process | 61 | LTB/CSF1R/IFIH1/GMFG/ARHGAP9/LRMP/PYCARD/CCDC88B/SMPD3/ZEB1/CORO1A/CCL4/OAS2/RAC2/HLA-F/MYD88/STAT1/HCLS1/FASLG/APBB1IP/RASGRP4/OSTF1/CEBPE/HMOX1/PILRA/HLA-DPB1/RASSF2/CMTM7/PTGER4/APOBEC3G/CTSD/PVRIG/DOCK2/SNX10/IFIT1/LGALS9/HLA-DRB4/ATP6V0C/CRTAM/BST2/SDC4/MX1/OAS1/IRS2/CD83/AIM2/NCKAP1L/CCR7/PRKCH/NLRP3/PMAIP1/RHOH/MMP1/NFKBID/KCNAB2/LPXN/RASGRP1/SELL/GPR171/ITGB2/PDE5A |
| GO:0002682 | regulation of immune system process | 39 | IFIH1/PYCARD/CCDC88B/SMPD3/ZEB1/CORO1A/CCL4/RAC2/HLA-F/MYD88/STAT1/HCLS1/HMOX1/PILRA/HLA-DPB1/RASSF2/PTGER4/APOBEC3G/PVRIG/IFIT1/LGALS9/HLA-DRB4/CRTAM/BST2/SDC4/IRS2/CD83/AIM2/NCKAP1L/CCR7/PRKCH/NLRP3/NFKBID/LPXN/RASGRP1/SELL/GPR171/ITGB2/PDE5A |
| GO:0046903 | secretion | 39 | CSF1R/IFIH1/GMFG/ARHGAP9/LRMP/PYCARD/SMPD3/CORO1A/SLC25A4/OAS2/CARD8/RAC2/HLA-F/MYO5A/OSTF1/HMOX1/PTGER4/TIAM1/CTSD/DOCK2/SNX10/LGALS9/ATP6V0C/CRTAM/BST2/SDC4/IRS2/AIM2/NCKAP1L/CCR7/MYOM1/NLRP3/ADRA2C/AQP9/KCNAB2/RASGRP1/SGK1/SELL/ITGB2 |
| GO:0006955 | immune response | 45 | LTB/CSF1R/IFIH1/GMFG/ARHGAP9/LRMP/PYCARD/CORO1A/CCL4/OAS2/RAC2/HLA-F/MYD88/STAT1/FASLG/APBB1IP/OSTF1/HMOX1/PILRA/HLA-DPB1/PTGER4/APOBEC3G/CTSD/PVRIG/DOCK2/IFIT1/LGALS9/HLA-DRB4/ATP6V0C/CRTAM/BST2/MX1/OAS1/CD83/AIM2/NCKAP1L/CCR7/PRKCH/NLRP3/NFKBID/KCNAB2/LPXN/RASGRP1/SELL/ITGB2 |
| GO:0045321 | leukocyte activation | 33 | GMFG/ARHGAP9/LRMP/PYCARD/CCDC88B/ZEB1/CORO1A/RAC2/HLA-F/APBB1IP/OSTF1/HMOX1/HLA-DPB1/CMTM7/PTGER4/CTSD/DOCK2/LGALS9/ATP6V0C/BST2/SDC4/IRS2/CD83/NCKAP1L/CCR7/NLRP3/RHOH/NFKBID/KCNAB2/RASGRP1/SELL/ITGB2/PDE5A |
| GO:0001775 | cell activation | 35 | GMFG/ARHGAP9/LRMP/PYCARD/CCDC88B/ZEB1/CORO1A/RAC2/HLA-F/APBB1IP/OSTF1/HMOX1/HLA-DPB1/CMTM7/PTGER4/CTSD/DOCK2/LGALS9/ATP6V0C/BST2/SDC4/IRS2/CD83/NCKAP1L/CCR7/PRKCH/NLRP3/ADRA2C/RHOH/NFKBID/KCNAB2/RASGRP1/SELL/ITGB2/PDE5A |
| GO:0002252 | immune effector process | 33 | IFIH1/GMFG/ARHGAP9/LRMP/PYCARD/CORO1A/OAS2/RAC2/HLA-F/STAT1/APBB1IP/OSTF1/HMOX1/PTGER4/APOBEC3G/CTSD/DOCK2/IFIT1/LGALS9/ATP6V0C/CRTAM/BST2/MX1/OAS1/AIM2/NCKAP1L/NLRP3/PMAIP1/NFKBID/KCNAB2/RASGRP1/SELL/ITGB2 |
| GO:0046649 | lymphocyte activation | 24 | PYCARD/CCDC88B/ZEB1/CORO1A/RAC2/HLA-F/APBB1IP/HLA-DPB1/CMTM7/PTGER4/DOCK2/LGALS9/BST2/SDC4/IRS2/CD83/NCKAP1L/CCR7/NLRP3/RHOH/NFKBID/RASGRP1/ITGB2/PDE5A |
| GO:0034097 | response to cytokine | 32 | LTB/CSF1R/IFIH1/PYCARD/SMPD3/ZEB1/CORO1A/CCL4/OAS2/CARD8/HLA-F/MYD88/STAT1/HCLS1/FASLG/HMOX1/HLA-DPB1/LAPTM5/SNX10/IFIT1/LGALS9/HLA-DRB4/BST2/MX1/OAS1/IRS2/AIM2/CCR7/TYMS/MMP1/CXCR6/ITGB2 |
| GO:0051049 | regulation of transport | 39 | CSF1R/IFIH1/PYCARD/SMPD3/CORO1A/CCL4/SLC25A4/OAS2/CARD8/RAC2/HLA-F/MYO5A/HCLS1/NTF3/HMOX1/ADRB2/PTGER4/TIAM1/CRYAB/DOCK2/LGALS9/CRTAM/BST2/SDC4/IRS2/AIM2/NCKAP1L/CCR7/MYBPC3/MYOM1/NLRP3/PMAIP1/ADRA2C/KCNAB2/CATSPER1/RASGRP1/SGK1/DMPK/ITGB2 |
| **Cellular Component** | |  |  |
| GO:0061702 | inflammasome complex | 4 | PYCARD/CARD8/AIM2/NLRP3 |
| GO:0098805 | whole membrane | 28 | LRMP/CORO1A/NECAP2/RAC2/HLA-F/MYD88/FASLG/EFHD2/HMOX1/ATP6V1A/ADRB2/HLA-DPB1/LAPTM5/PPP2R2B/CTSD/SNX10/HLA-DRB4/ATP6V0C/BST2/ADRA1B/SDC4/NCKAP1L/PMAIP1/GPR137B/KCNAB2/SELL/DMPK/ITGB2 |
| GO:0000323 | lytic vacuole | 17 | LRMP/PYCARD/HLA-F/MYO5A/FASLG/ATP6V1A/ADRB2/HLA-DPB1/LAPTM5/CTSD/HLA-DRB4/ATP6V0C/BST2/SDC4/GPR137B/RASGRP1/TCN2 |
| GO:0005764 | lysosome | 17 | LRMP/PYCARD/HLA-F/MYO5A/FASLG/ATP6V1A/ADRB2/HLA-DPB1/LAPTM5/CTSD/HLA-DRB4/ATP6V0C/BST2/SDC4/GPR137B/RASGRP1/TCN2 |
| GO:0072559 | NLRP3 inflammasome complex | 3 | PYCARD/CARD8/NLRP3 |
| GO:0005773 | vacuole | 17 | LRMP/PYCARD/HLA-F/MYO5A/FASLG/ATP6V1A/ADRB2/HLA-DPB1/LAPTM5/CTSD/HLA-DRB4/ATP6V0C/BST2/SDC4/GPR137B/RASGRP1/TCN2 |
| GO:0098588 | bounding membrane of organelle | 30 | ST8SIA4/GIMAP1/LRMP/PYCARD/GALNT6/SMPD3/CORO1A/NECAP2/RAC2/HLA-F/MYD88/ATP6V1A/ADRB2/HLA-DPB1/LAPTM5/PPP2R2B/CTSD/SNX10/HLA-DRB4/ATP6V0C/BST2/NCKAP1L/NLRP3/PMAIP1/GPR137B/KCNAB2/RASGRP1/SELL/DMPK/ITGB2 |
| GO:0044437 | vacuolar part | 14 | LRMP/PYCARD/HLA-F/FASLG/ATP6V1A/HLA-DPB1/LAPTM5/CTSD/HLA-DRB4/ATP6V0C/BST2/SDC4/GPR137B/TCN2 |
| GO:0044459 | plasma membrane part | 32 | SLC16A7/LRMP/SLC46A1/CORO1A/SLC25A4/HLA-F/FASLG/APBB1IP/HMOX1/ATP6V1A/ADRB2/HLA-DPB1/LAPTM5/GPR34/TIAM1/CRYAB/HLA-DRB4/BST2/ADRA1B/SDC4/CD83/NCKAP1L/PLXNB1/GPR137B/ADRA2C/AQP9/RHOH/KCNAB2/CATSPER1/SELL/CXCR6/ITGB2 |
| GO:0031090 | organelle membrane | 36 | ST8SIA4/OSBPL3/GIMAP1/LRMP/PYCARD/GALNT6/SMPD3/CORO1A/SLC25A4/NECAP2/RAC2/HLA-F/MYD88/ATP6V1A/ADRB2/HLA-DPB1/LAPTM5/PPP2R2B/CTSD/SNX10/HLA-DRB4/ATP6V0C/BST2/ADRA1B/MX1/NCKAP1L/TYMS/NLRP3/PMAIP1/GPR137B/KCNAB2/RASGRP1/SELL/ALOX5AP/DMPK/ITGB2 |
| **Molecular Function** | |  |  |
| GO:0098772 | molecular function regulator | 28 | LTB/GMFG/ARHGAP9/PYCARD/CCL4/CARD8/RAC2/FASLG/RASGRP4/NTF3/DENND2D/ADRB2/CMTM7/TIAM1/PPP2R2B/DOCK2/BST2/NCKAP1L/MYBPC3/PLXNB1/SMAP2/RHOH/KCNAB2/RASGEF1B/RASGRP1/SGK1/ALOX5AP/DMPK |
| GO:0042802 | identical protein binding | 28 | CSF1R/IFIH1/PYCARD/CORO1A/CCL4/CARD8/MYO5A/MYD88/STAT1/GIMAP2/CEBPE/HMOX1/ADRB2/APOBEC3G/CRYAB/BST2/SDC4/MX1/AIM2/MYBPC3/MYOM1/TYMS/NLRP3/ADRA2C/RASSF3/RASGRP1/HSPB6/GSTM5 |
| GO:0001730 | 2'-5'-oligoadenylate synthetase activity | 2 | OAS2/OAS1 |
| GO:0005345 | purine nucleobase transmembrane transporter activity | 2 | SLC25A4/AQP9 |
| GO:0008047 | enzyme activator activity | 11 | GMFG/ARHGAP9/PYCARD/CARD8/DOCK2/NCKAP1L/MYBPC3/PLXNB1/SMAP2/RASGRP1/ALOX5AP |
| GO:0042803 | protein homodimerization activity | 16 | CSF1R/PYCARD/CORO1A/CARD8/STAT1/CEBPE/HMOX1/ADRB2/APOBEC3G/CRYAB/BST2/MYOM1/TYMS/ADRA2C/RASGRP1/HSPB6 |
| GO:0005088 | Ras guanyl-nucleotide exchange factor activity | 6 | RASGRP4/DENND2D/TIAM1/DOCK2/RASGEF1B/RASGRP1 |
| GO:0032813 | tumor necrosis factor receptor superfamily binding | 4 | LTB/MYD88/STAT1/FASLG |
| GO:0030234 | enzyme regulator activity | 16 | GMFG/ARHGAP9/PYCARD/CARD8/RAC2/PPP2R2B/DOCK2/BST2/NCKAP1L/MYBPC3/PLXNB1/SMAP2/RHOH/RASGRP1/ALOX5AP/DMPK |
| GO:0015205 | nucleobase transmembrane transporter activity | 2 | SLC25A4/AQP9 |
| **KEGG** |  |  |  |
| hsa05164 | Influenza A | 12 | IFIH1/PYCARD/SLC25A4/OAS2/MYD88/STAT1/FASLG/HLA-DPB1/HLA-DRB4/MX1/OAS1/NLRP3 |
| hsa05323 | Rheumatoid arthritis | 7 | LTB/ATP6V1A/HLA-DPB1/HLA-DRB4/ATP6V0C/MMP1/ITGB2 |
| hsa05416 | Viral myocarditis | 5 | RAC2/HLA-F/HLA-DPB1/HLA-DRB4/ITGB2 |
| hsa05152 | Tuberculosis | 8 | CORO1A/MYD88/STAT1/HLA-DPB1/CTSD/HLA-DRB4/ATP6V0C/ITGB2 |
| hsa04621 | NOD-like receptor signaling pathway | 8 | PYCARD/OAS2/CARD8/MYD88/STAT1/OAS1/AIM2/NLRP3 |
| hsa05162 | Measles | 7 | IFIH1/OAS2/MYD88/STAT1/FASLG/MX1/OAS1 |
| hsa05330 | Allograft rejection | 4 | HLA-F/FASLG/HLA-DPB1/HLA-DRB4 |
| hsa05332 | Graft-versus-host disease | 4 | HLA-F/FASLG/HLA-DPB1/HLA-DRB4 |
| hsa04145 | Phagosome | 7 | CORO1A/HLA-F/ATP6V1A/HLA-DPB1/HLA-DRB4/ATP6V0C/ITGB2 |
| hsa04940 | Type I diabetes mellitus | 4 | HLA-F/FASLG/HLA-DPB1/HLA-DRB4 |

**Supplementary Table S4.** Complete list of DEGs from three algorithms via CytoHubba plug-in.

| Degree | Betweenness | Closeness | Intersection |
| --- | --- | --- | --- |
| STAT1 | STAT1 | ITGB2 | STAT1 |
| ITGB2 | RAC2 | STAT1 | RAC2 |
| RAC2 | CCL4 | CCL4 | CCL4 |
| CCL4 | ITGB2 | SELL | ITGB2 |
| MX1 | SELL | RAC2 | SELL |
| MYD88 | SLC46A1 | MYD88 | NCKAP1L |
| SELL | NCKAP1L | CSF1R | CSF1R |
| IFIH1 | HMOX1 | CCR7 | MYD88 |
| LAPTM5 | CSF1R | LAPTM5 | BCL2A1 |
| CSF1R | TYMS | DOCK2 | MX1 |
| IFIT1 | MYD88 | HCLS1 | CCR7 |
| OAS1 | BCL2A1 | MX1 | NLRP3 |
| HCLS1 | MX1 | IFIH1 | DOCK2 |
| DOCK2 | HLA-F | ARHGAP9 | HCLS1 |
| CCR7 | TCN2 | BCL2A1 | ARHGAP9 |
| BST2 | CCR7 | CORO1A | LAPTM5 |
| OAS2 | ALOX5AP | IFIT1 | CORO1A |
| ARHGAP9 | NLRP3 | FASLG |  |
| CORO1A | TIAM1 | NLRP3 |  |
| NCKAP1L | DOCK2 | NCKAP1L |  |
| NLRP3 | HCLS1 | BST2 |  |
| APBB1IP | ARHGAP9 | CD83 |  |
| PARVG | GZMK | ALOX5AP |  |
| SAMD9L | FASLG | APBB1IP |  |
| PARP12 | PRKCH | OAS1 |  |
| AIM2 | GIMAP4 | GMFG |  |
| HLA-F | HLA-DPB1 | OAS2 |  |
| BCL2A1 | TES | LGALS9 |  |
| GMFG | LAPTM5 | PARVG |  |
| TIAM1 | CORO1A | GZMK |  |


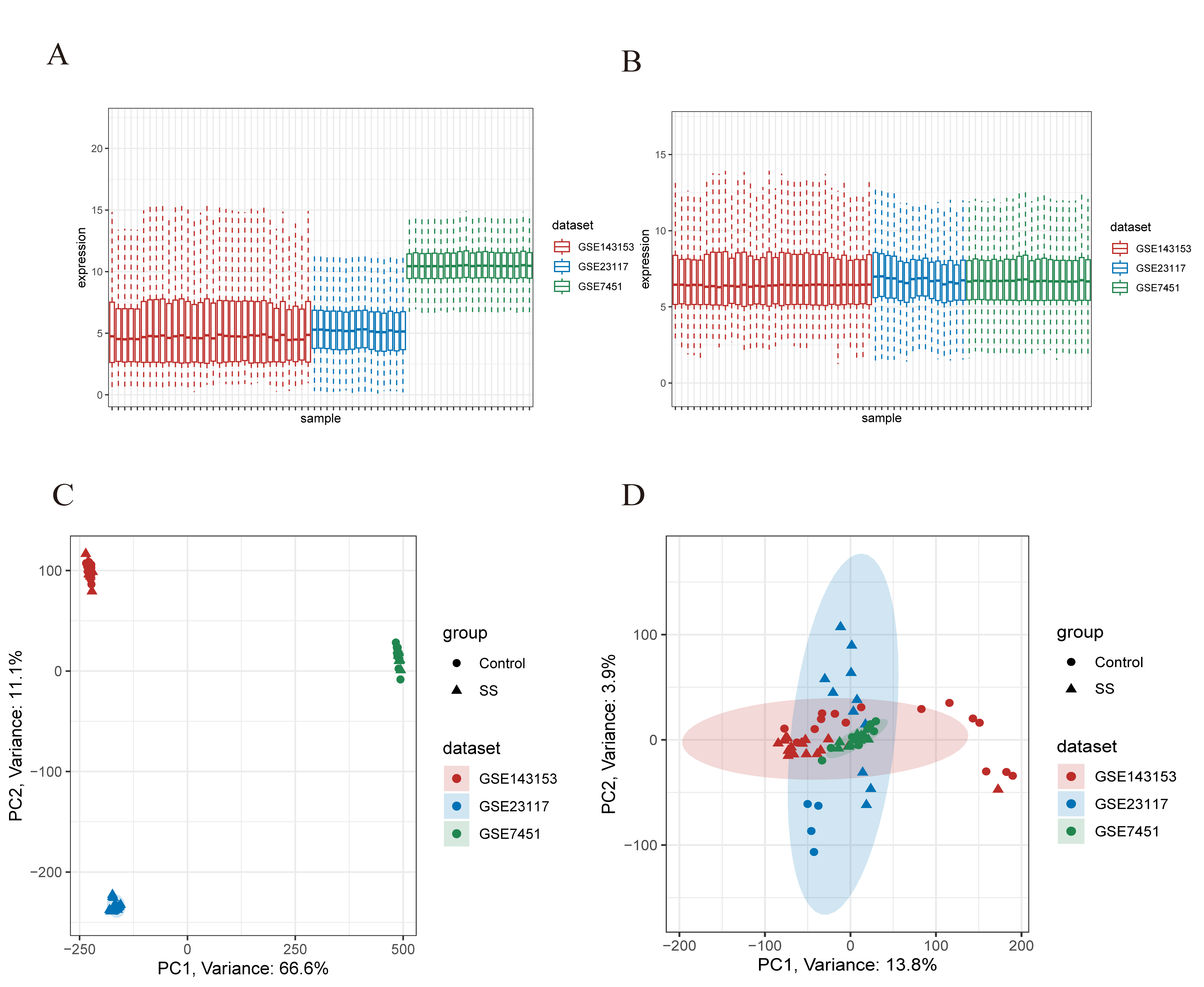


**Supplementary Figure S1.** Merged GSE7451, GSE23117, and GSE143153 datasets with removal of unwanted variations and batch effects.

(A) Box plot before removing batch effects.

(B) Box plot after removing batch effects

(C) PCA plot before removing batch effects.

(D) PCA plot after removing batch effects.


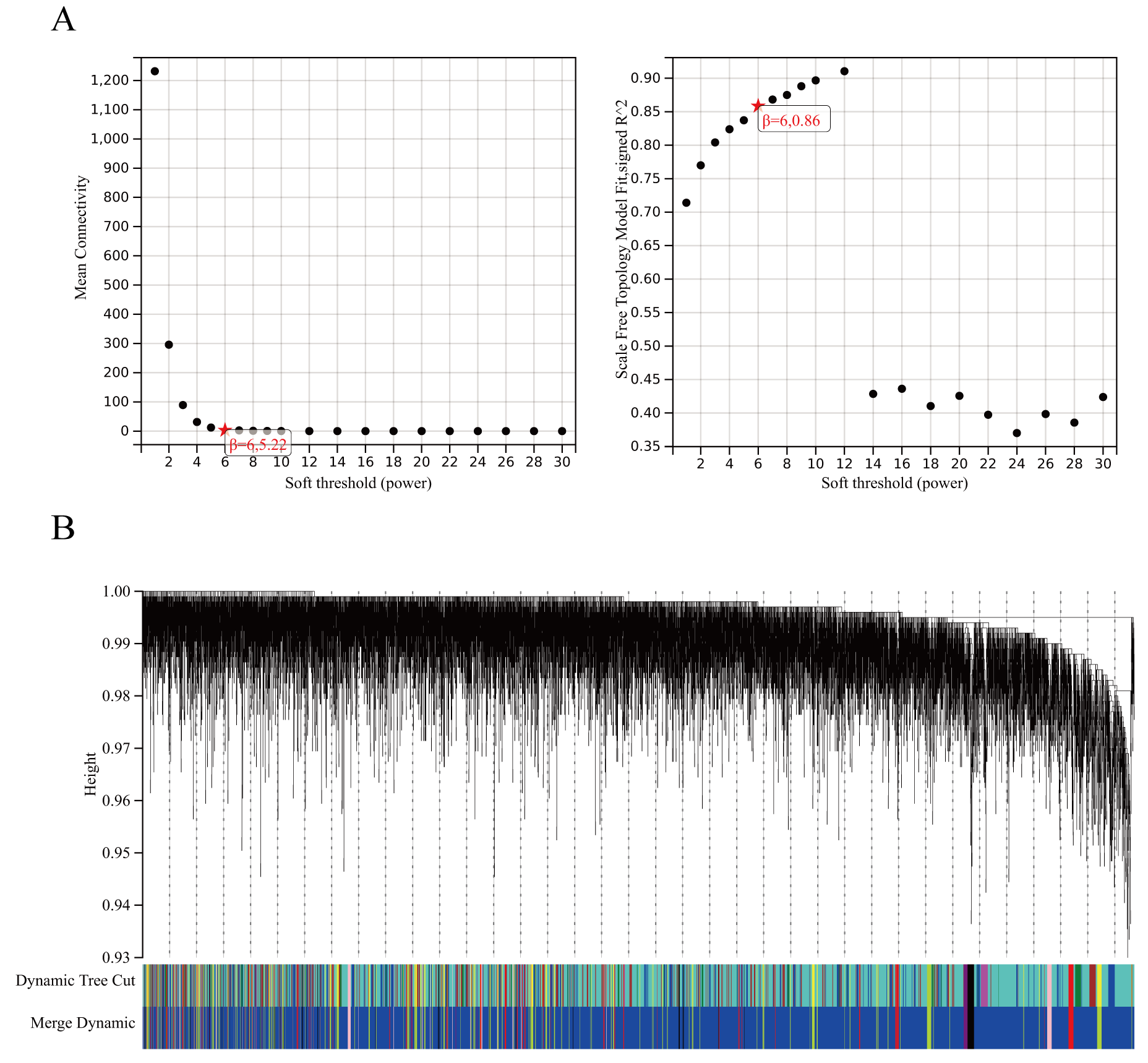


**Supplementary Figure S2.** Soft thereshold selection and gene cluster tree via WGCNA of SS

1. The soft threshold selection. β=6 was choose as the most appropriate threshold.
2. Gene cluster tree of different modules.


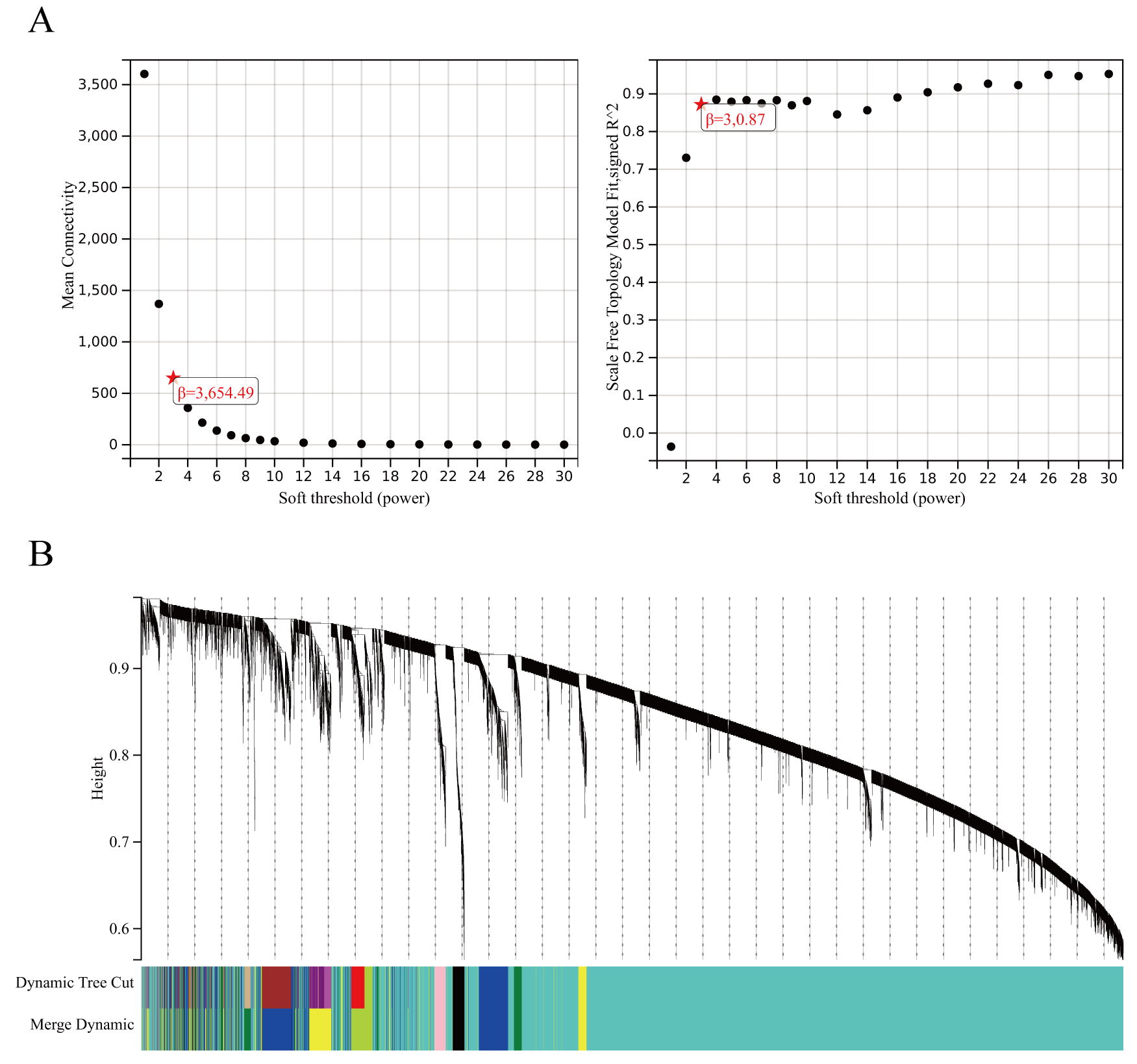


**Supplementary Figure S3.** Soft thereshold selection and gene cluster tree via WGCNA of PA

1. The soft threshold selection. β=3 was choose as the most appropriate threshold.
2. Gene cluster tree of different modules.


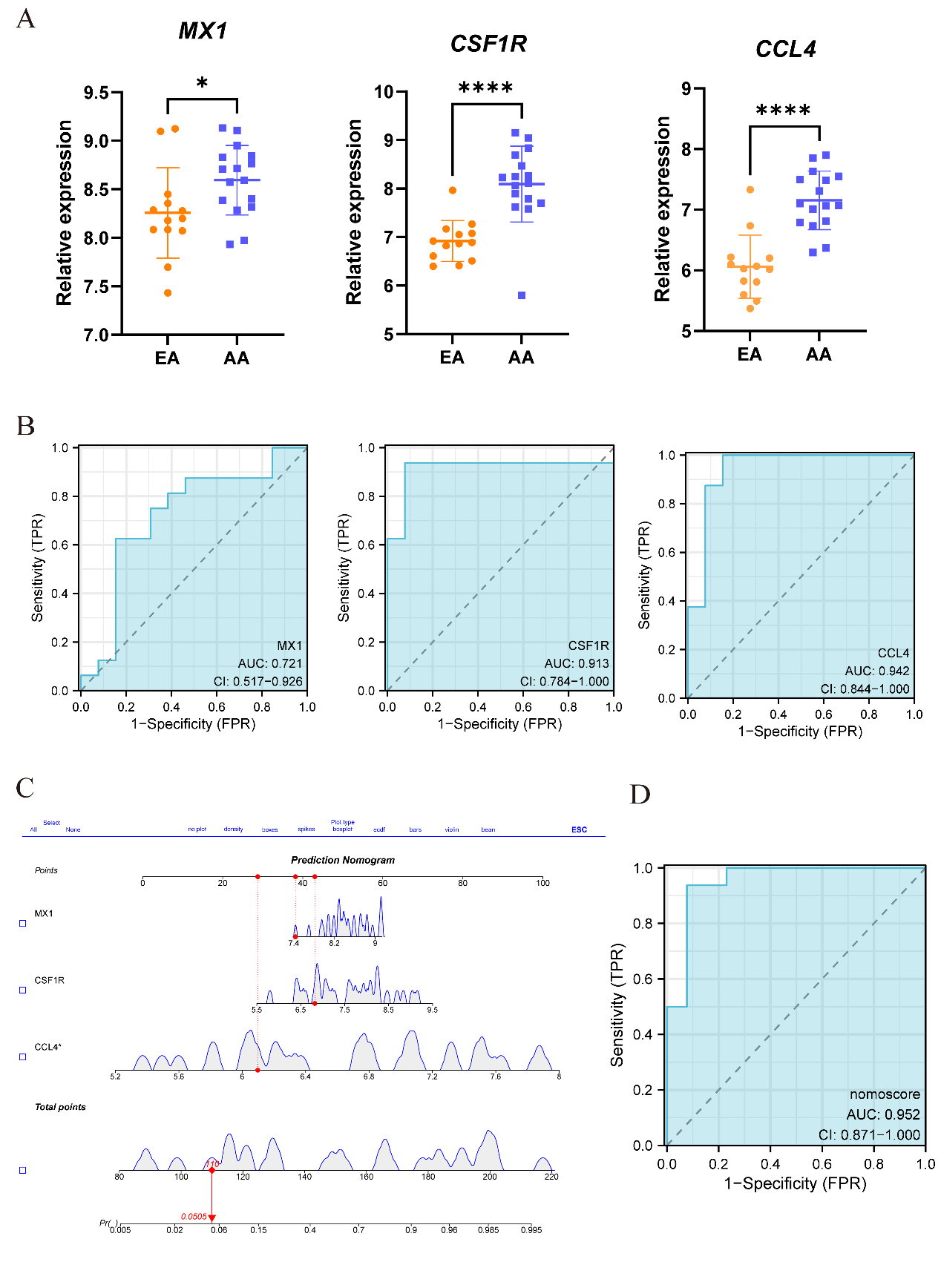


**Supplementary Figure S4**. The diagnostic value of candidate biomarkers and the nomogram construction in the validation dataset GSE28829.

1. The expression difference of the three genes between AA and EA. *P < 0.05, ****P < 0.0001.
2. The diagnostic value of the three genes and the nomogram in AA from the ROC curve. Each panel displayed the AUC under the curve and 95% CI.

(C) The construction of a diagnostic nomogram employing the three genes aims to enhance the diagnosis of AA.

(D) The ROC curve for the nomogram is depicted in patients with AA.


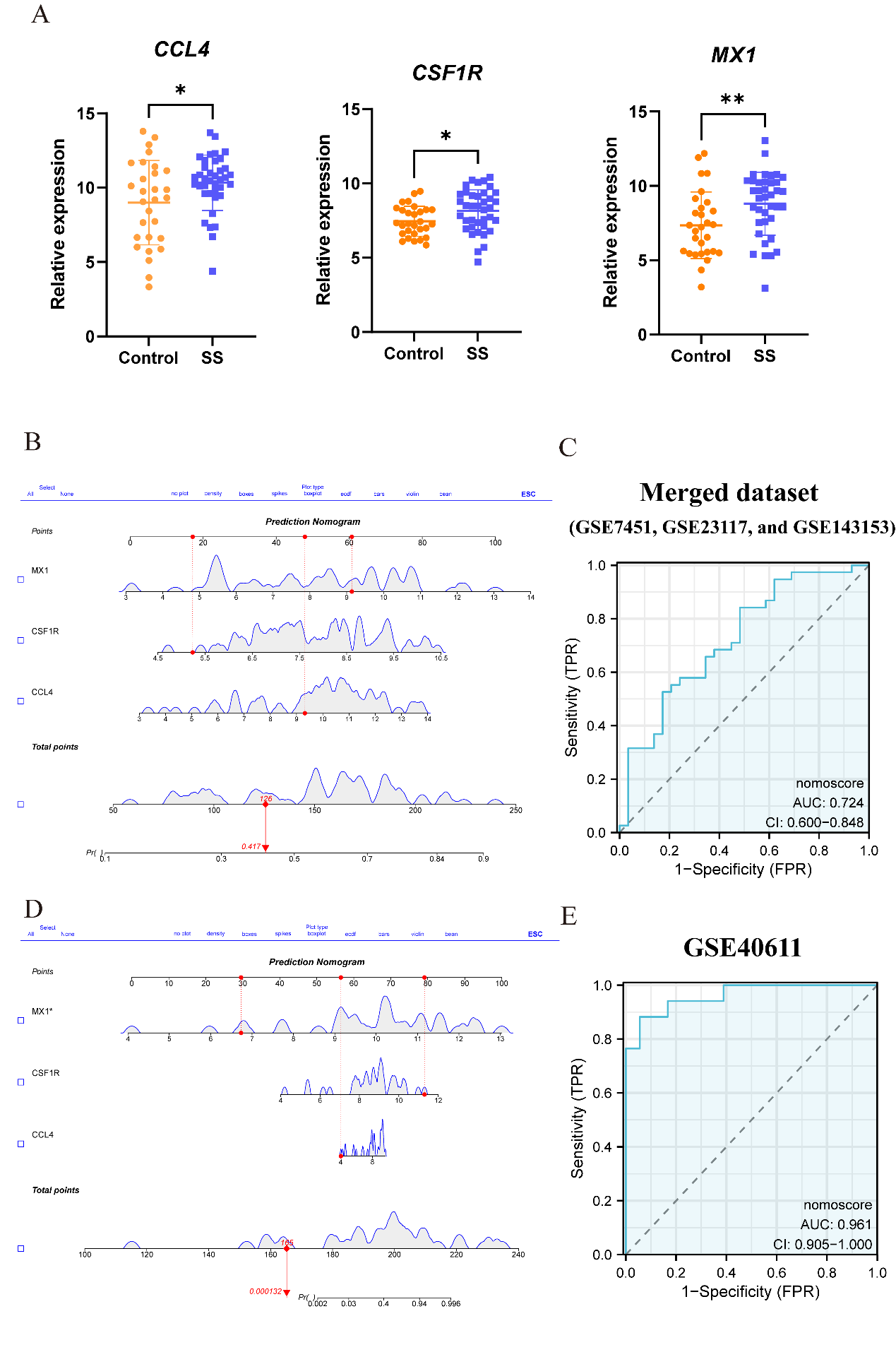


**Supplementary Figure S5.** Evaluation of ROC and nomogram construction in

SS and the clinical diagnostic value of the nomogram in the validation dataset.

(A) Differential expression is observed in three genes when comparing the and control groups (* p < 0.05, ** p < 0.01).

(B) The construction of a diagnostic nomogram employing the three genes aims to enhance the diagnosis of SS.

(C) The ROC curve for the nomogram is depicted in patients with SS.

(D) The construction of a diagnostic nomogram employing the three genes aims to enhance the diagnosis of SS in the validation dataset GSE40611.

(E) The ROC curve for the nomogram is plotted in SS patients from the validation dataset GSE40611.


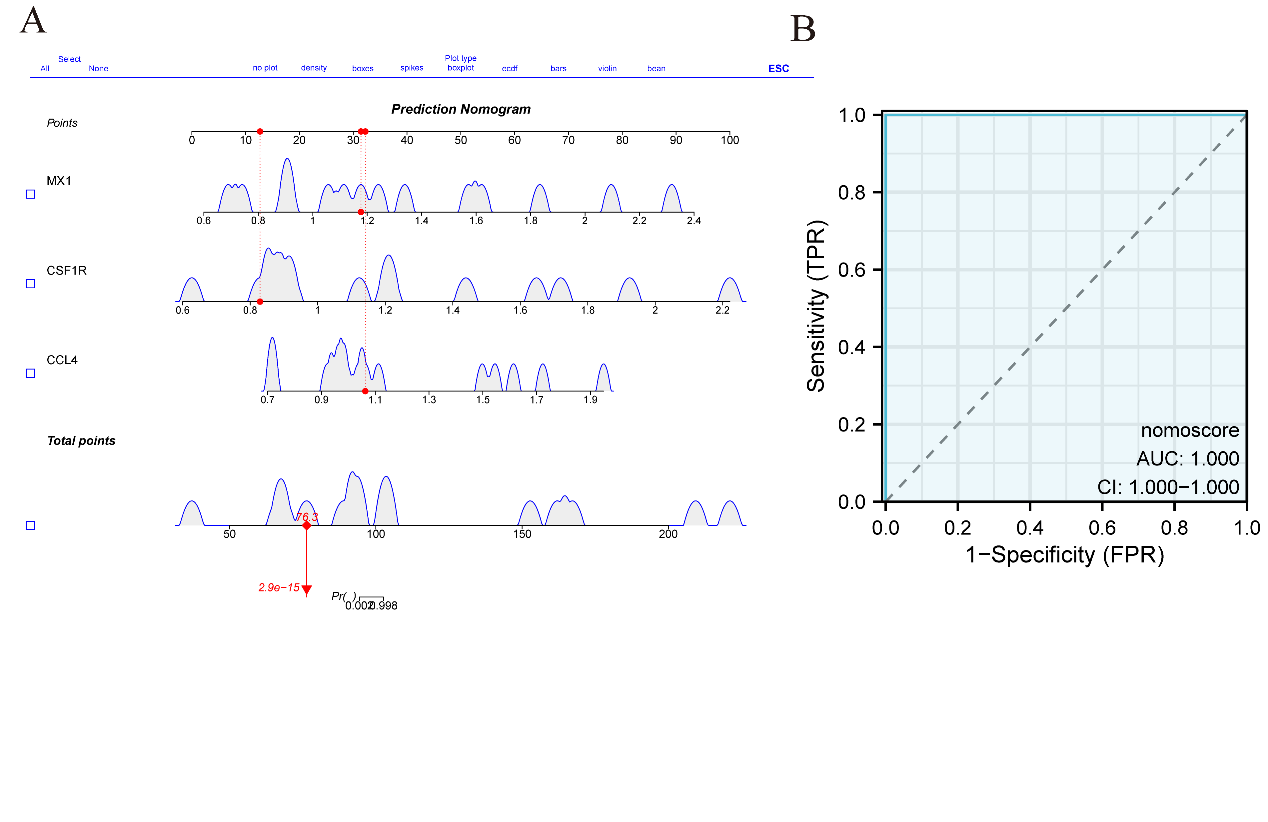


**Supplementary Figure S6.** Nomogram construction and evaluation of ROC in clinical sample.

(A) A nomogram was developed using three externally validated DEGs. Each DEG corresponds to a score on the nomogram. The final score was determined by adding the scores for each DEG.

(B) ROC curve of the nomogram revealed DEGs with prominent predictive value for PA in patients with SS.


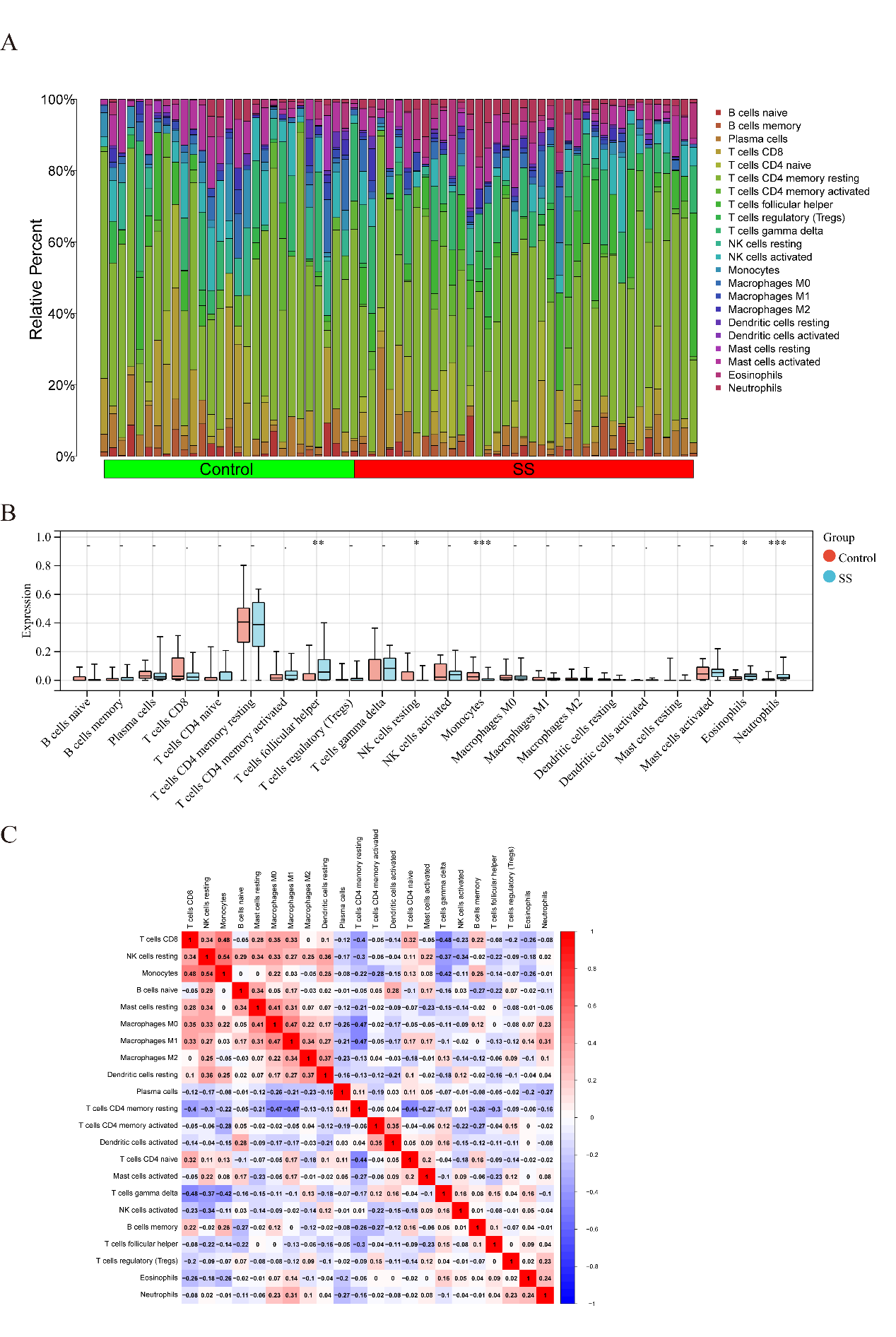


**Supplementary Figure S7**. Immunological alterations between control and SS groups.

(A) A bar plot representing the distribution of immune cells among different samples.

(B) A boxplot comparing the expression of immune cells between the SS and control groups, with statistical significance denoted as *p < 0.05, **p < 0.01, ***p < 0.001.

(C) A heatmap illustrating the correlations among various immune cells implicated in the pathogenesis of SS.


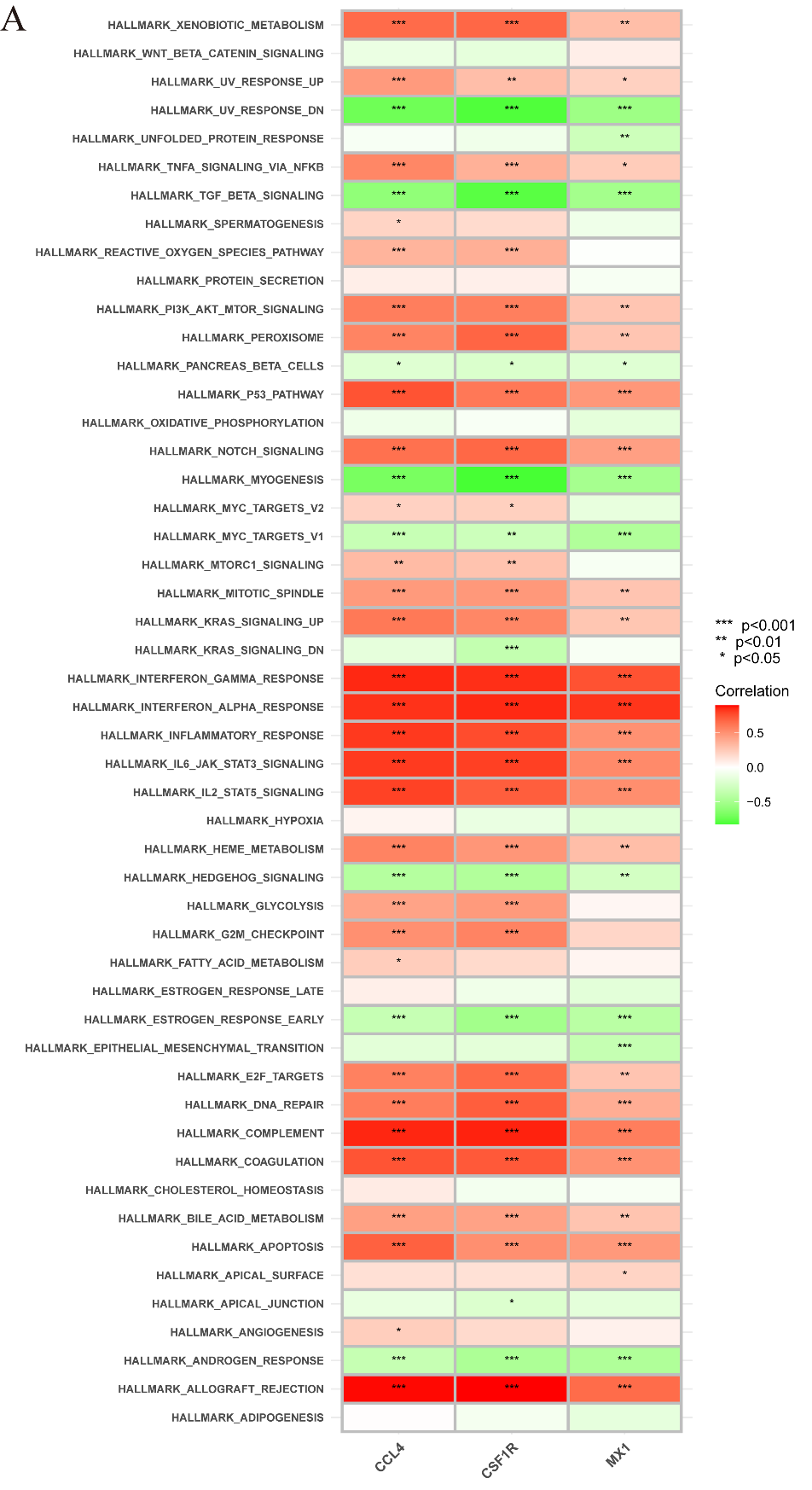


**Supplementary Figure S8.** Correlation analysis of the three candidate biomarkers with hallmark gene sets via ssGSEA
